# Intersections of Climate Change with Food Systems, Nutrition, and Health: An Overview and Evidence Map

**DOI:** 10.1016/j.advnut.2024.100274

**Published:** 2024-07-15

**Authors:** Thalia Morrow Sparling, Claudia Offner, Megan Deeney, Philippa Denton, Kristin Bash, Rachel Juel, Susan Moore, Suneetha Kadiyala

**Affiliations:** 1 Department of Population Health, London School of Hygiene and Tropical Medicine, London, United Kingdom; 2 Independent; 3 Population Health, School of Medicine and Population Health, The University of Sheffield, Sheffield, United Kingdom; 4 Health Sciences, School of Business, The University of York, York, United Kingdom

**Keywords:** climate mitigation, climate adaptation, agriculture, food, nutrition, research synthesis, Evidence and Gap Map, review

## Abstract

Scientific research linking climate change to food systems, nutrition, and nutrition-related health (FSNH) has proliferated, showing bidirectional and compounding dependencies that create cascading risks for human and planetary health. Within this proliferation, it is unclear which evidence to prioritize for action and which research gaps, if filled, would catalyze the most impact. We systematically searched for synthesis literature (i.e., reviews) related to FSNH published after 1 January, 2018. We screened and extracted characteristics of these reviews and mapped them in an interactive Evidence and Gap Map (EGM) supplemented by expert consultation. Eight hundred forty-four synthesis reports met inclusion criteria (from 2739 records) and were included in the EGM. The largest clusters of reports were those describing climate impacts on crop and animal-source food production and emissions from such (86%). Comparatively few reports assessed climate change impacts on nutrition-related health or food manufacture, processing, storage, and transportation. Reports focused on strategies of climate adaptation (40%), mitigation (29%), both (19%), or none (12%). Only 1 quarter of reports critically evaluated equity (25%), and fewer reports suggested that changes to equity and equitable practices would alter climate-FSNH dynamics (6%). The expert consultation mirrored the results of the EGM and contextualized findings further. This novel map describes a wide research landscape linking climate change to FSNH. We identified 4 key research gaps: *1*) research on whole food systems or postharvest elements; *2*) research evaluating relationships between climate change and nutrition-related health outcomes, especially among vulnerable populations; *3*) promising methods (and additional data required) that can i) identify inflection points or levers for intervention, ii) incorporate complex dynamics and characterize trade-offs, iii) be understood and applied in context-specific, localized ways for decision making; and *4*) research undertaken through interdisciplinary collaborations that enables producing and translating evidence to action, especially those that inherently consider coproduction and fairness.

**Statements of significance**

Many reviews and primary studies exist detailing the varied relationships between climate change and food systems, nutrition, and health. However, to our knowledge, this is the first systematic overview and analysis of this body of research as a whole, and we offer an interactive tool Evidence and Gap Map with which to explore it.

## Introduction

Climate change presents an urgent planetary crisis. The climate is rapidly changing due to human activities, primarily the emission of greenhouse gases (GHGs), such as carbon dioxide, methane, and nitrous oxide. These emissions have led to an unprecedented increase in global temperatures, alterations in precipitation patterns, rising sea levels, and more frequent extreme weather events [1].

At the same time, our approach to food systems has not realized the goal of providing global populations with adequate food and good nutrition, which disproportionately affects those living in low- and middle-income countries and contexts [2]. More than a fifth of countries are experiencing high-food prices [3], and almost 2.5 billion people live without reliable access to sufficient, safe, and nutritious food. There is limited or no progress in reducing global undernutrition, overweight, and diet-related chronic diseases [4]. By 2030, our current trajectory predicts a world of vast social inequities, in which 575 million people still live in extreme poverty amidst the many reverberating impacts of surpassing 1.5°C of global warming [3].

Food systems are simultaneously contributing to and acutely suffering from climate change and environmental degradation [5]. From production to waste disposal, food systems are responsible for >30% of GHG emissions [6], 70% of freshwater use, and 80% of deforestation [7]; they are the primary driver of biodiversity loss and a leading contributor to chemical and plastic pollution [[8], [9], [10]]. That a third of the world’s food is lost or wasted exacerbates impacts on the environment and population health further still[11].

Climate change impacts the composition and nutrient availability of certain foods, leading to changes in nutritional quality. For instance, elevated carbon dioxide levels can decrease the protein and mineral content in crops, changing the nutritional value of foods, as well as crop yields and quality [12]. This, in turn, affects food supply and prices. Climate conditions and extreme events disrupt supply chains, leading to shortages, price volatility, potential food insecurity, and increases in emissions to offset disruption [2]. Health effects linked to climate change, such as heat stress, food-borne illness, infectious disease prevalence, and mental health, are compounded by consequences of food systems and nutritional status, such as cyclically reducing capacity in the workforce [13].

These challenges present critical opportunities: the ways that climate change is dynamically linked to agriculture, food systems, nutrition, and nutrition-related health (FSNH) can create compounding detriments but, in turn, could also offer potential for synergistic action. Sustainable food systems have the potential to ensure availability, accessibility, affordability, and demand for safe and nutritious foods produced and consumed in ways that protect and restore the natural environment [14]. Scientific research on climate change-FSNH intersections has proliferated, especially in the last 5 y, generating an overwhelming amount of literature on this topic [1]. However, a holistic view of our current knowledge is missing. Equally important is recognizing areas that require more research to advance our understanding of these complex relationships enough to prioritize actions effectively. For instance, research on climate adaptation and mitigation strategies, considerations of equity, and methodological innovations have become increasingly relevant in providing solutions to climate-FSNH challenges, but many gaps exist within these themes [[15], [16], [17]].

We mapped research linking climate change to FSNH to systematically characterize this wide intersection and clarify the most urgent areas for future research and action. An Evidence and Gap Map (EGM), along with expert consultation and analysis of both, provides an in-depth view of this research and coalesces recommendations for future directions. We intend this EGM and analysis to be useful for both academics and the wider community of practice working on these topics.

## Methods

Given the breadth of these 2 intersecting topics, we chose to include only synthesis articles in the last 5 y. We followed a combined approach of creating an EGM from a systematic literature review [18], also drawing from elements of a rapid realist review approach, namely a qualitative review involving content experts [19]. We included literature only from the last 5 y to focus on the most up-to-date synthesis, which we considered the most appropriate approach for drawing recommendations for future research. Furthermore, because we included reviews, these naturally cover literature from a much longer period. Additional considerations about the strengths and limitations of this methodology can be found in Supplemental Methods 1**.**

### Search strategy

We conducted a systematic search of titles from 3 published literature databases, Web of Science, Scopus, and Medline, from 1 January, 2018 until 31 January, 2023.

Our search string included climate change-related terms, such as global warming, GHG, extreme weather, and rising sea levels/temperatures. We included a range of terms related to agriculture and food production, food systems terms, nutrition terms, and nutrition-related health terms. To ensure that all relevant synthesis literature was included, we searched both title and abstract for “review” and “overview.” The full search strategy is specified in Supplemental Methods 2.

We also searched published grey literature to capture content and key recommendations from sectors and actors less likely to publish peer-reviewed literature. We searched Global Index Medicus, the World Bank, 3IE, and CGIAR (formerly the Consultative Group on International Agricultural Research) databases, adapting the search terms to each repository as possible. All search hits were imported into EPPI Reviewer software, where duplicates were removed. Following the consultation, if key reports suggested by experts on this topic met our inclusion criteria, they were also added to the EPPI Reviewer results and screened and coded in line with the rest. A full list of resources suggested by experts is offered in Supplemental Results 1.

### Eligibility

We included reviews if they were published in English from 1 January, 2018 until 31 January, 2023 from anywhere in the world. We considered most types of synthesis articles (i.e., any type of review, including subject-matter and literature reviews, overviews, peer-reviewed book chapters, meta-analyses, or policy reviews) to be eligible for inclusion. We excluded primary analyses or case study papers. We also excluded literature types that were unpublished reports or nonreviewed book chapters, in addition to conference abstracts, theses, errata, protocols, and entire books. If a report included a literature search that was solely the foundation for a primary modeling analysis, this was not considered a review. Any generalizable population was included. Reports about the environment, plants, or animals that were not explicitly linked to food production were excluded.

Climate change was divided into 2 main domains: GHG emissions (e.g., nitrogen, carbon, methane, and combustion-related air pollution) and anthropogenic weather changes (e.g., rising temperatures, extreme storms, changing precipitation patterns, and droughts). Reports falling within these domains were considered for inclusion. We excluded reviews examining unrelated emissions, environmental, or climate outcomes (e.g., fMRI tomography emissions, built environments, and environmental contamination) that were not explicitly linked to climate change. Studies reviewing the impacts of isolated extreme events (e.g., Hurricane Katrina) were also excluded. Iteratively, reviews covering related anthropogenic drivers of climate change were grouped and coded as an additional domain, even if these were not part of the search strategy.

FSNH was divided into 5 main domains: agro-environments (e.g., soil, pests, microbes, agroforestry, and agricultural waste); primary food production (e.g., crops, livestock, aquaculture, and agricultural policy); postharvest systems (e.g., supply chains, food processing, food loss/waste, food environments, and packaging or labeling); food security and diets; and nutrition-related health outcomes (e.g., malnutrition, diabetes, cardiovascular diseases, and communicable diseases). We excluded ecology, forestry, or mineral reviews that were not explicitly linked to agriculture, food, or nutrition. Papers focusing on non-nutrition-related health outcomes or exposures (e.g., respiratory or cardiovascular health unlinked to nutrition) were also excluded.

Finally, we excluded any reviews that did not clearly explain links between ≥1 component of climate change to ≥1 component of FSNH (i.e., if climate change and FSNH were treated as separate subjects). A full list of the inclusion/exclusion criteria can be found in Supplemental Methods 3.

### Screening and study selection

A team of experienced researchers was trained to screen records. All records were double-screened on title and abstract in pairs that included a senior researcher (either TS or CO). Patterns and disagreements were regularly discussed, and guidance was updated accordingly. All eligible records were then double-screened with the same guidance using full text. For papers not freely available or accessible through institutional subscription, we contacted corresponding authors to request full text.

### Data coding and analysis

A single researcher extracted data for each included report, which was then reviewed by a senior researcher. We used a custom form in EPPI Reviewer to extract relevant characteristics, including FSNH domains, climate change themes, strategy (e.g., climate adaptation – the process of adapting to a changing climate or climate mitigation – the process of reducing the drivers of climate change, year published, type of review, sociodemographic population, setting (geographic, income level, and agroecologic zones), the hypothesized direction of the studied relationship (climate change driving FSNH outcomes, the reverse or bidirectional), engagement with themes of equity, the mechanisms studied or proposed change (including interventions, tools, research methods, or metrics), and key recommendations as stated by authors. A full description of these categories and accompanying rationale is provided in Supplemental Methods 4A–C.

We used several agriculture, nutrition, and health and food systems frameworks, such as the high-level panel of expert reports, to organize the framework (i.e., the rows and columns of the EGM), especially for the FSNH domains [20]. Because of thematic overlap in most frameworks, we organized this section iteratively, first deductively according to frameworks and then iteratively with a test sample for the subdomains. Characteristics of reports such as setting and population of interest were determined a priori, whereas mechanisms of change and author recommendations were created inductively as a method of “horizon scanning” (and because there are no established frameworks for these). Most reviews covering interrelated themes were coded in multiple categories and thus can be identified regardless of the prevailing approach of the reader.

### Expert consultation

To enrich research recommendations and triangulate results from the literature search, we conducted a qualitative expert consultation of 18 people from 15 organizations. The expert consultants were selected as leading researchers and practitioners in the climate change and FSNH domains. Experts were asked to highlight any landmark papers fitting the inclusion criteria, their subjective assessment of research or evidence gaps, and suggestions for future research, particularly in regard to tools, methods, and metrics still needed. We used a thematic content analysis to synthesize responses and compare them to the EGM results.

We used the expert consultation to integrate additional themes into the EGM coding structure, especially on the key recommendations, including codes to identify papers discussing COVID-19, migration or displacement, and indigenous knowledge. Records already coded were re-assessed for these themes, and we performed a second coding of key recommendations on all included reports. These are important themes because they represent interacting vulnerabilities and issues of equity and exclusion. Users can select these codes as filters to find research on these themes as long as the review fits within the overarching inclusion criteria (i.e., these themes were not part of the inclusion criteria/search strategy but coded if they fit the broader remit.

### Evidence and gap map

All reports that met inclusion criteria were mapped into an EGM using standard methods [18]. The EGM is a fixed framework of FSNH domains (in rows) and climate change domains (in columns), each with collapsible subcategories. Each cell is segmented into 4 color-coded bubbles, scaled proportionally to the number of studies in each group. The user can click on cells to open a bibliography of reports at that thematic intersection. A filter system can also be used to select studies with more specific characteristics of interest.

## Results

### Search and screening results

**FIGURE 1:**
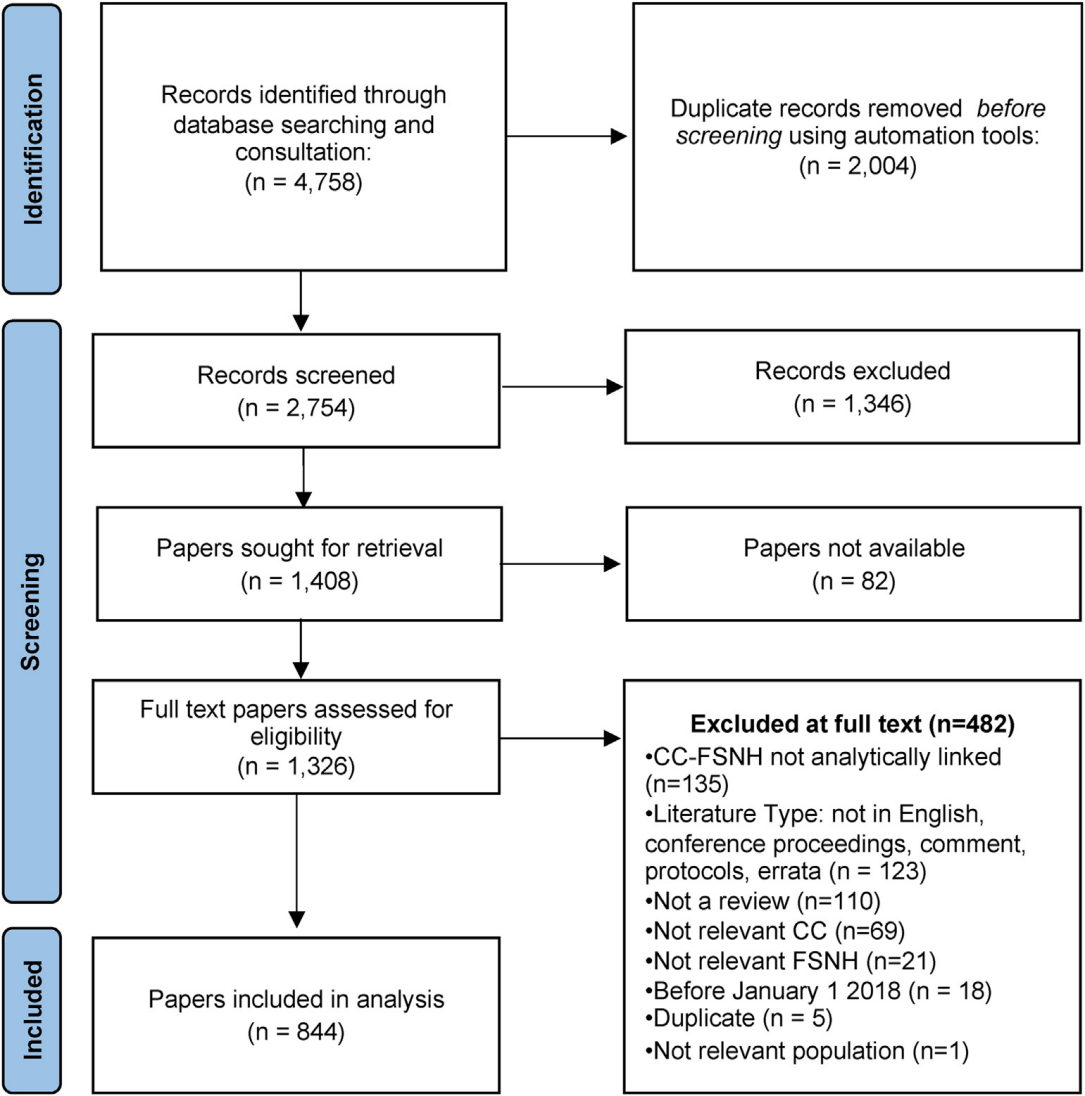
PRISMA flowchart of included studies. CC, climate change; FSNH, food systems, nutrition, and nutrition-related health; PRISMA, preferred reporting items for systematic reviews and meta-analyses.

The study selection process is shown in the PRISMA flowchart (Figure 1). We screened 2754 unique records on title and abstract and 1326 on full text. Eight hundred forty-four studies met the inclusion criteria and were mapped onto our EGM using a fixed framework of climate change and FSNH categories (https://www.anh-academy.org/climate-change-egm). The cells in the EGM are segmented by climate change strategies: climate adaptation (orange), climate mitigation (yellow), both (green), and none (blue).

Climate change and FSNH thematic analyses are presented below in order of the prevalence of reports covering each domain. The results tabulated here forth often do not add up to the total number of reports included, because many studies are coded on multiple domains, populations, and settings.

#### Aspects of climate change

Almost two-thirds of reports focused on changes in weather patterns (*n* = 500), with more than half of these (*n* = 264/500) discussing nonspecific weather changes. Of the specific weather phenomena examined, ambient temperature was most prominent (35%, *n* = 177/500), followed by drought (27%, *n* = 137/500) and precipitation patterns (16%, *n* = 78/500).

Half of the reviews examined GHGs and related emissions (*n* = 427), mostly in general (48%, *n* = 207/427), but many focused on specific GHGs, particularly carbon (43%, *n* = 185/427) and nitrogen (25%, *n* = 107/427). Almost a fifth covered both GHGs and weather (18%, *n* = 155). Some studies did not specify which aspects of climate change they were discussing (11%, *n* = 95).

Although not explicitly part of our search strategy, many of the included studies examined other anthropogenic drivers indirectly linked to climate change (34%, *n* = 289). Of these, the most prominent groups were environmental pollutants (35%, *n* = 101/289), energy (28%, *n* = 81/289), environmental degradation (25%, *n* = 72/241), and salinization (24%, *n* = 70/289).

#### Aspects of FSNH

Reports on farm-level food production comprised the most saturated of the FSNH domains, consisting of 87% of reviews (*n* = 734). Within this, crops (65%, *n* = 480/734) and animal-source foods (ASFs; 28%, *n* =204/734) were the most covered, with much of each category focusing on general crop or livestock production rather than specific crops or animals. Crop and ASF themes were not mutually exclusive, and many papers examined both (14%, *n* = 117). Aquaculture was the least reviewed category within food production (9%, *n* = 66/734).

Reports on agro-environments were the second most prominent FSNH domain, accounting for 57% of reviews (*n* = 481). Within this, the major themes were soil (52%, *n* = 251/481), water systems (37%, *n* = 179/481), biodiversity/agroecology (29%, *n* = 141/481), land use (20%, *n* = 96/481), and disease, pathogens, and pests (20%, *n* = 96/481).

Postharvest systems reports were the third most populated FSNH domain (30% of all records, *n* = 256). Most of these reports focus on food environments (36%, *n* = 93/256) or food systems policy (31%, *n* = 79/256), but other prominent themes were food waste or loss (24%, *n* = 62/256) and economics (24%, *n* = 61/256). Few reports focused on themes such as food supply chains and transport (*n* = 42), packaging and labeling (*n* = 19), food safety (*n* = 19), or food prices and expenditure (*n* = 17), albeit several of these subdomains are part of larger food environments frameworks (in addition to being commonly discussed in relationship to consumer choice and diets), and thus are doubly coded under food environments, as appropriate.

Food security and diets were a central theme for 17% of records (*n* = 140), the overwhelming majority of which focused on food security (91%, *n* = 128/140), followed by diets (47%, *n* = 66/140). Food security was covered by reviews both as an aspect of food systems (including pillars aligned to food environments such as availability and utilization) and an aspect of consumption or nutrition. Thus, there was an overlap between these subdomains in the coding. Although considered part of diets, research on infant and child feeding made up only 5% of reports (*n* = 7/140).

Nutrition and nutrition-related health reports comprised the least populous FSNH domain, containing 12% of the records (*n* = 103). Half of these corresponded to noncommunicable diseases (51%, *n* = 53/103), followed by forms of malnutrition (46%, *n* = 47/103) and communicable diseases (33%, *n* = 34/103). Only 21 reviews focused on overweight or obesity, whereas 38 reviews considered underweight, micronutrient malnutrition, or growth faltering in early life.

**FIGURE 2:**
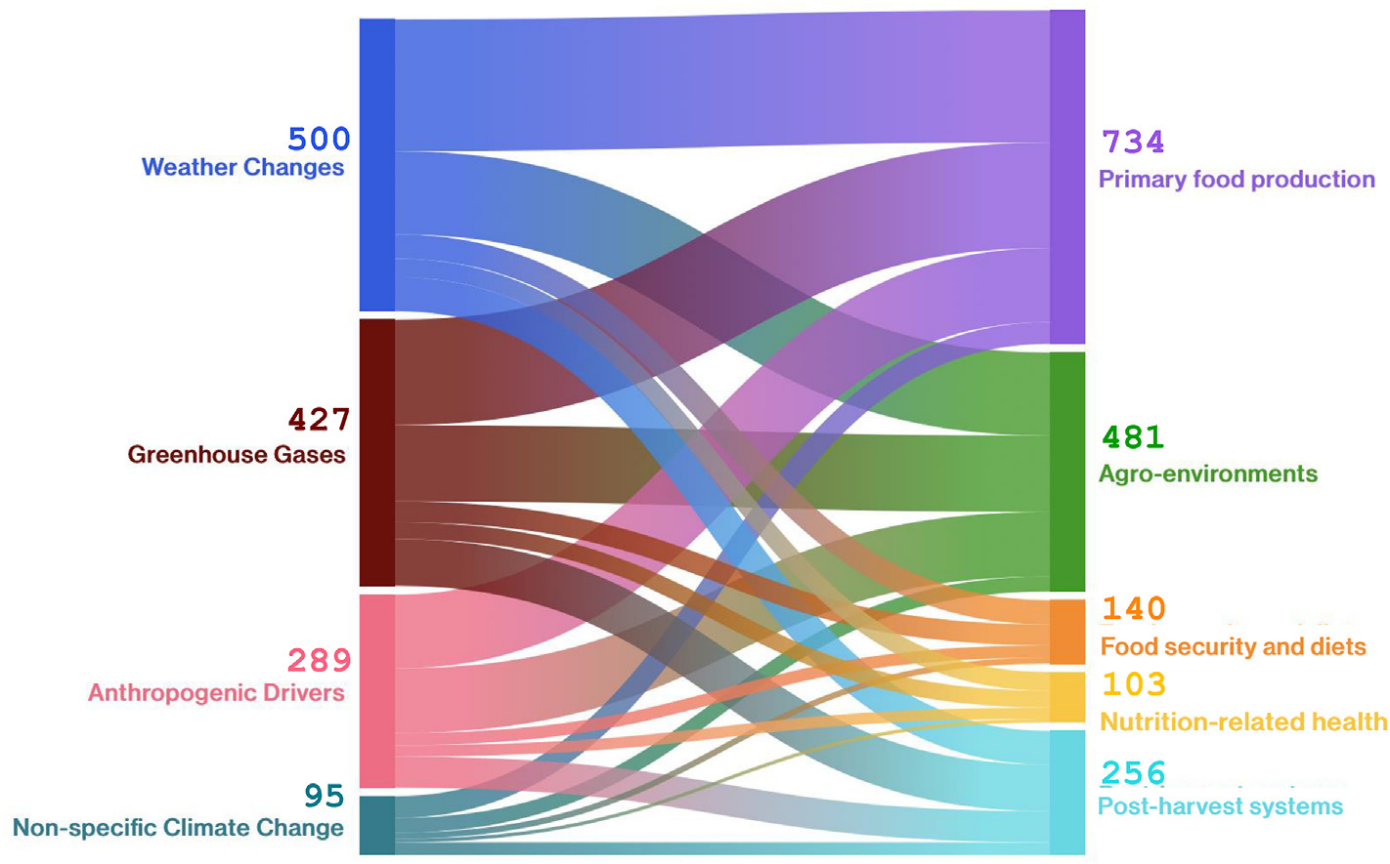
Sankey diagram of the number of studies linking climate change to food systems, nutrition, and nutrition-related health (FSNH). Major categories of climate change on the left are proportionally linked to corresponding major groups of FSNH on the right.

The links made between climate change and FSNH domains are illustrated in Figure 2. The largest group of reports linked weather changes to primary food production (54%, *n* = 455), followed by reports linking GHGs to the same (43%, *n* = 364), and weather changes to agro-environments (34%, *n* = 287). The fewest reports linked nutrition-related health or food security and diets to different domains of climate change, particularly nonspecific climate change (reports where authors generally spoke about climate change but did not specify the aspects) (nutrition-related health: 1%, *n* = 12; food security and diets 2%, *n* = 21) and anthropogenic drivers of climate change (nutrition-related health: 5%, *n* = 39; food security and diets 2%, *n* = 21).

**FIGURE 3:**
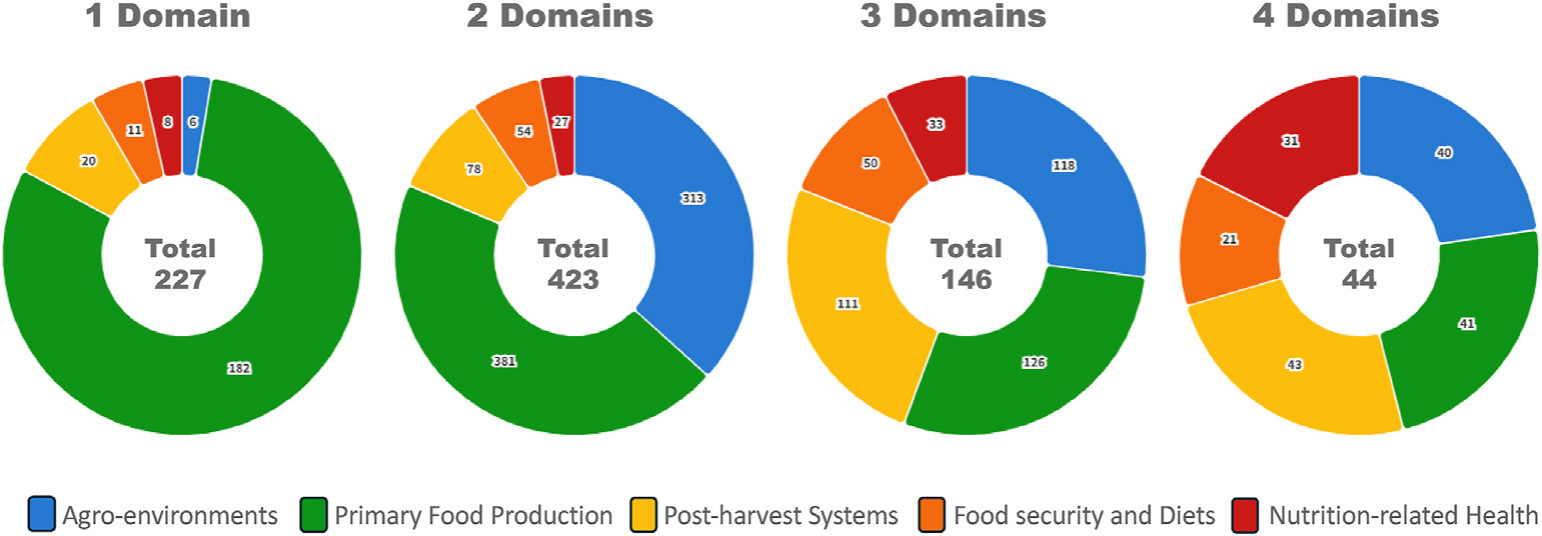
Distribution of reviews crossing 5 food systems, nutrition, and nutrition-related health (FSNH) domains (agro-environments, primary food production, postharvest systems, food security and diets, and nutrition-related health). Donut diagrams show the number of reviews covering the following: *1*) single FSNH domains (far left), *2*) 2 FSNH domains only (middle left right), *3*) 3 FSNH domains only (middle right), and *4*) 4 domains only (far right). There were 4 reports covering all 5 FSNH domains. The color blocks of each ring are proportional to the number of reviews focused on those domains. The number of reviews within each band of the rings is not additive.

For linkages between FSNH domains (i.e., parts of food systems, food security and diets, nutrition, and nutrition-related health), 227 reviews only focused on 1 of the FSNH domains. Many of the reviews covered >1 FSNH domain (73%), especially those in “adjacent” or most closely related categories of FSNH (e.g., agriculture environments and on-farm food production). Most covered 2 FSNH domains (*n* = 416). Fewer incorporated >2 FSNH domains, especially from the most “distant” or different ends of the FSNH pathway. For instance, 35% (*n* = 298) of records examined both agro-environments and on-farm food production domains, whereas 8% (*n* = 70) examined these 2 domains as well as domains from postharvest food systems. Only 4 reports included aspects of all 5 FSNH domains. The links made between climate change and FSNH domains are illustrated in Figure 2 and further broken down in Figure 3.

#### Type of reviews included

**FIGURE 4:**
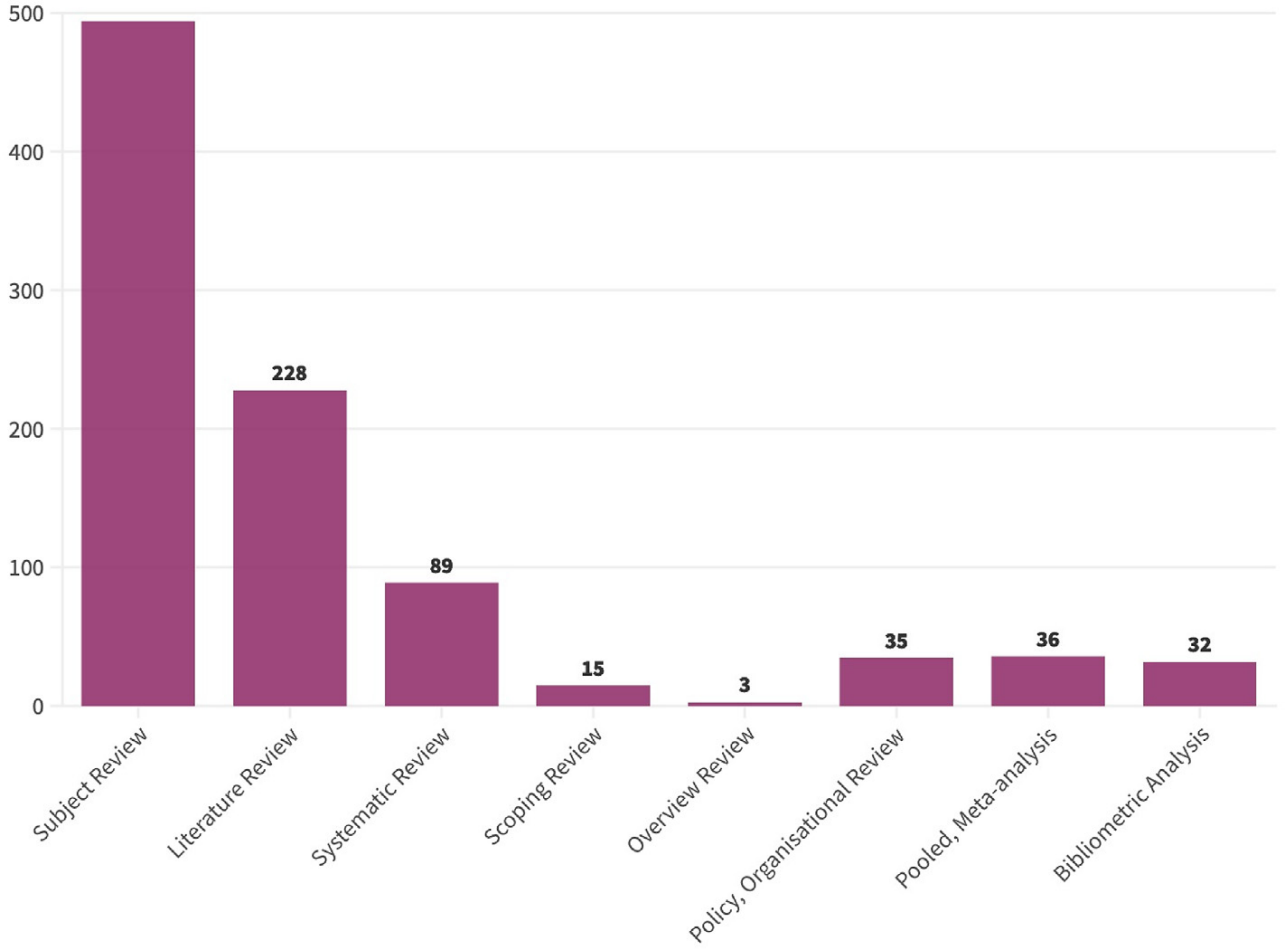
Distribution of included reports by type of review.

We identified several different types of research synthesis (Figure 4)*.* More than half of the included reviews were subject or content reviews with no stated methodological approach (*n* = 494). Just over a quarter of reports were nonsystematic literature reviews (*n* = 228), and 10% were systematic reviews that generally followed PRISMA or RepOrting standards for Systematic Evidence Synthesis in environmental research (ROSES) reporting guidelines (*n* = 89). Four percent were policy or organizational reviews, and another 4% offered a meta-analysis. Scoping reviews and bibliographic analyses were less common.

#### Settings, populations, and trends over time

**FIGURE 5:**
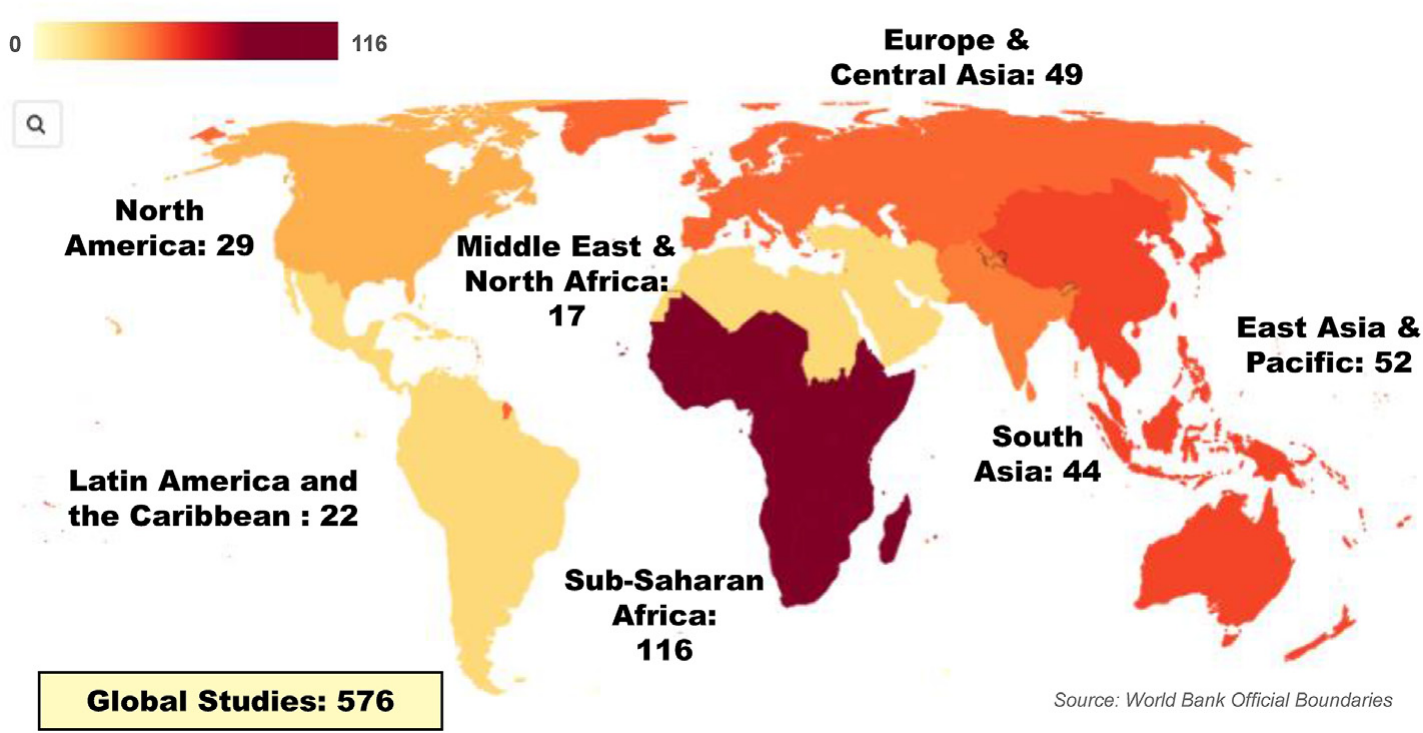
Choropleth map showing the saturation of reviews focusing on specific regions.

Most reports drew from diverse global literature (68%, *n* = 576) without limiting their geographic scope to specific countries or regions (Figure 5). Some took a regional focus, such as sub-Saharan Africa (14%, *n* = 116), East Asia and Pacific (6%, *n* = 52), Europe and Central Asia (6%, *n* = 49), South Asia (5% *n* = 44), North America (4%, *n* = 29), Latin America and the Caribbean (3%, *n* = 22), and Middle East and North Africa (2%, *n* = 17). Others specifically examined economic settings, such as low- and middle-income countries (13%, *n* = 110) and the European Union (4%, *n* = 32). A few specifically examined agro-environmental zones, such as arid/semi-arid (5%, *n* = 40) and tropical (4%, *n* = 33) regions. Small island developing states were the economic grouping with the least focus (1%; *n* = 9). Of the country-specific papers, India, the United States, and China were the most prominent (3%, *n* = 23; 2%, *n* = 21; 2%, *n* = 18, respectively).

Of the reviews on human populations (*n* = 608), 77% were general (*n* = 468/608). Occupation, which mainly consisted of farmers/smallholder populations, accounted for 17% of these reviews (*n* = 102/608). Reviews focusing on age groups, genders, race/ethnicity/culture, or place of residence were rare, as were those focusing only on children. Almost 40% of reports did not focus on a human population and rather focused on other relevant FSNH components, such as environments, animals, or plants (*n* = 236).

**FIGURE 6:**
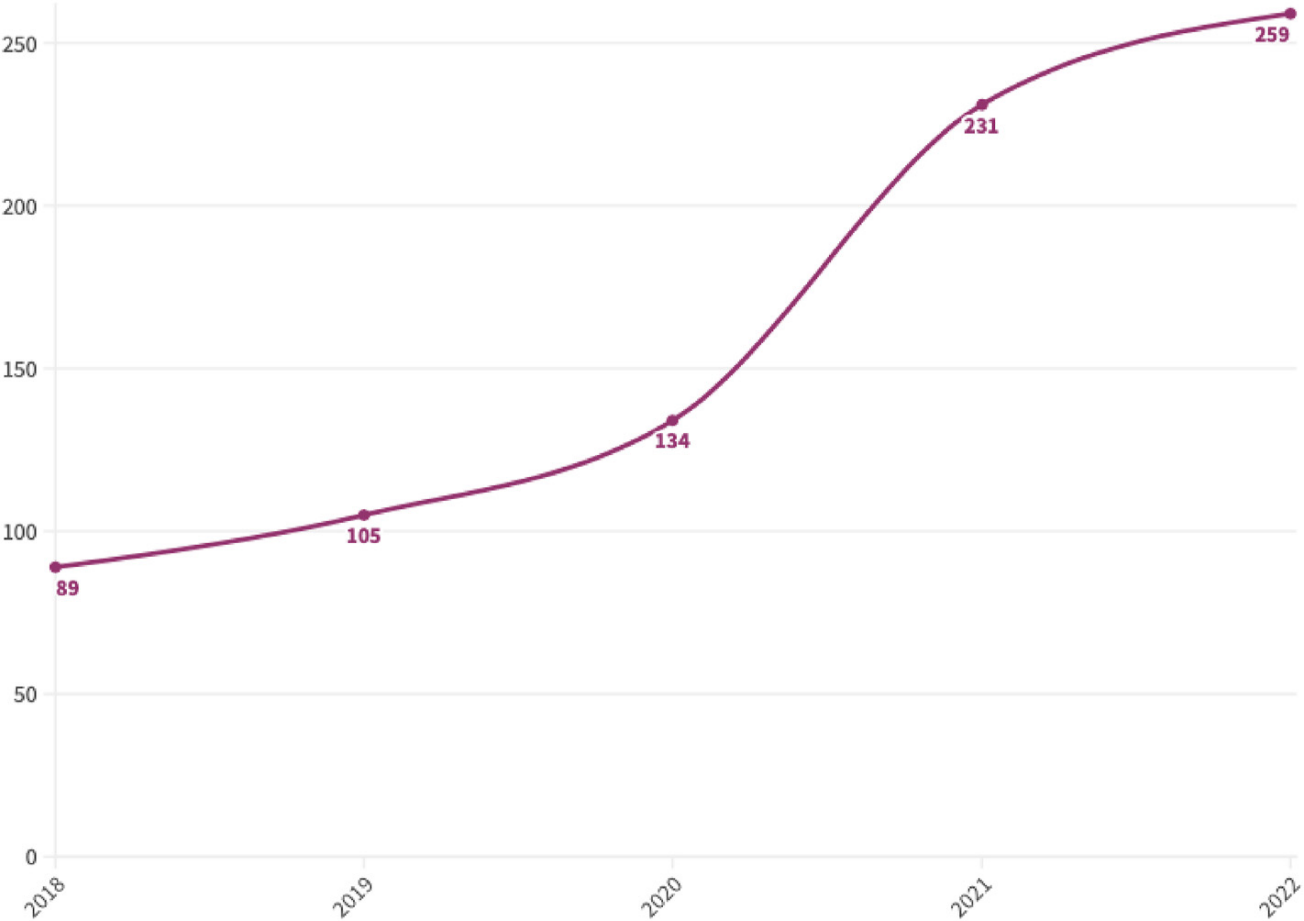
Number of reviews over time linking climate change to food systems, nutrition, and nutrition-related health between 1 January, 2018 and 31 December 2022. Between 1 and 31 January 2023 there were 26 reviews published, which are not included in the graph.

Our analysis shows that the overarching body of research syntheses linking climate change to FSNH has steadily grown by almost 200% in the past 5 y, from 89 in 2018 to 259 in 2022 (Figure 6). As we concluded our search at the beginning of 2023, the number of these studies annually will most likely continue to increase, marking a continued interest in this cross-section of fields.

#### Equity

Three-quarters of the reviews did not critically evaluate equity, meaning that they did not discuss equity as a driver of climate change-FSNH relationships or equitable outcomes related to either. Of the papers that did engage critically with issues of equity (*n* = 214), socioeconomic status was the most discussed (62%, *n* = 132/214), followed by place of residence (32%, *n* = 68/214), gender/sex (29%, *n* = 63/214), and occupation (26%, *n* = 55/214). The focus on occupation mirrors the prevalence of reviews focusing on farmer and smallholder populations.

#### Directionality: the hypothesized relationship between climate change and FSNH

**FIGURE 7:**
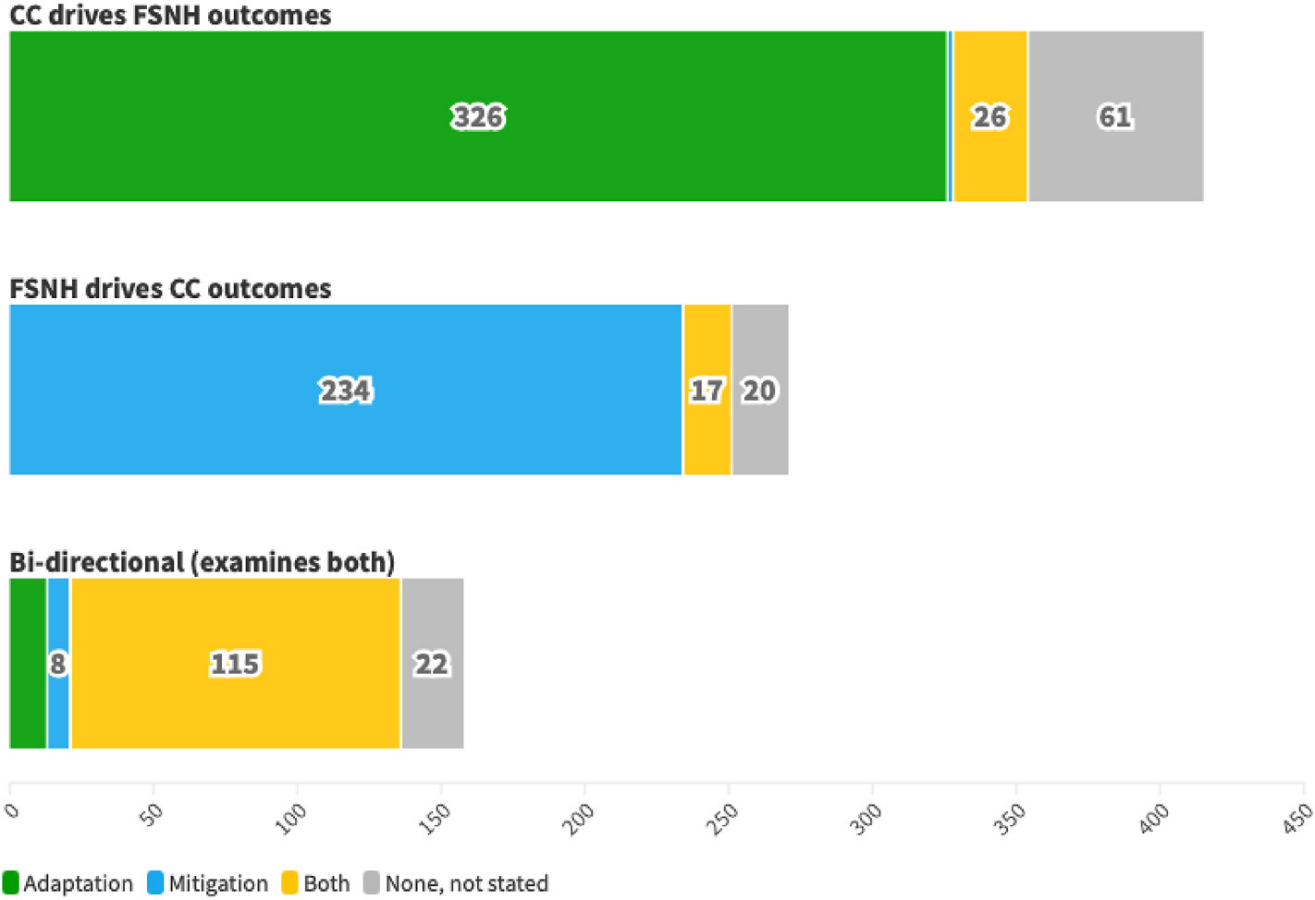
Direction of relationship (described or hypothesized) between climate change (CC) and food systems, nutrition, and health (FSNH), segmented by climate adaptation and mitigation strategies.

Forty-nine percent of all records examined climate change as an exposure driving FSNH outcomes, 32% examined FSNH as an exposure driving climate change outcomes, and 19% assessed bidirectional relationships. Figure 7 summarizes these relationships.

#### Climate change adaptation and mitigation strategies

Forty-three percent of reviews discussed climate adaptation strategies, whereas 25% evaluated climate mitigation strategies. More than 23% discussed both adaptation and mitigation strategies, usually through dynamic mechanisms of change, such as climate-smart agriculture or dietary decision support. Around 9% of reports discussed neither—these were typically descriptive reviews lacking solution-oriented discussions.

#### Mechanisms of change

Most reports (89%) described some mechanism of change to achieve better outcomes, such as policies, technologies, programs, or interventions (i.e., ways to change outcomes) or understand relationships better through research innovation (e.g., frameworks, methods, or metrics, also including technology). Categories of mechanisms of change included policy, technology, knowledge, attitudes, and practices (i.e., ways to shift knowledge, attitudes, or practices; see Supplemental Methods 3A for definitions) and research-focused mechanisms (i.e., tools, methods, metrics, and data required to produce better research).

Mechanisms of changing knowledge, attitudes, and practices were the most commonly discussed (67%, *n* = 564), with practices being most prevalent (90%, *n* = 509/564). The largest of this category were agro-environmental practices (45%, *n* = 227/564), crop practices (24%, *n* = 124/564), water practices (23%, *n* = 115/564), and animal practices (21%, *n* = 106/564). Technology mechanisms were the second largest grouping (51%, *n* = 433). They were mostly saturated by biotechnology (79%, *n* = 344/433), particularly genetics and genetically modified organisms (e.g., gene editing and engineering and climate-resilient breeding) (37%, *n* = 128/433), fertilizers or pesticides (31%, *n* = 107/433), and microbiomes (17%, *n* = 58/433). Policy and governance change mechanisms were not often put forth 16% (*n* = 137).

**FIGURE 8:**
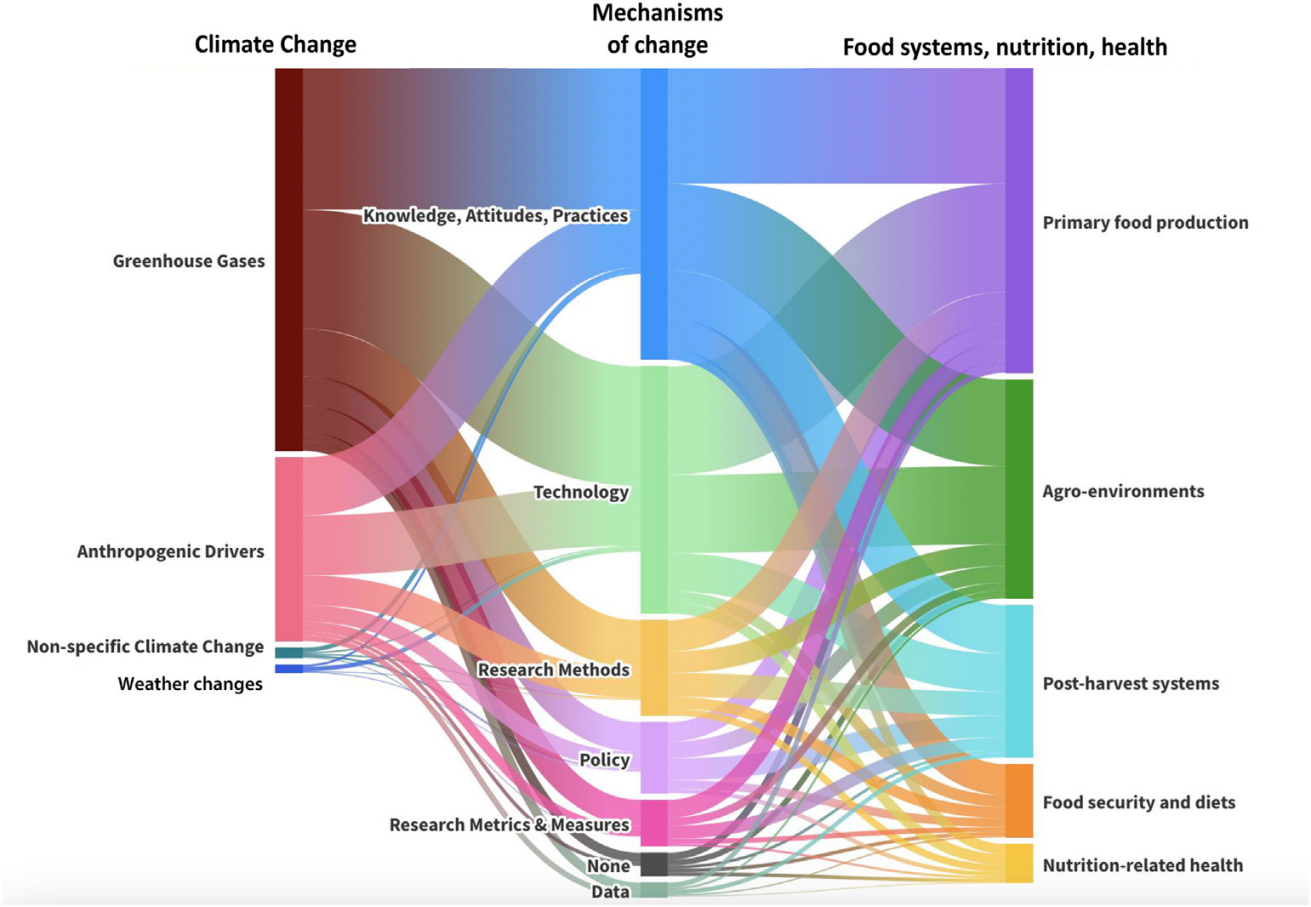
Sankey alluvial flow diagram showing how reviews characterize different types of mechanisms of change (ways to change the relationship, in the middle) between food systems, nutrition, and nutrition-related health (FSNH) domains (agro-environments, primary food production, postharvest systems, food security and diets, and nutrition-related health) on the left, connected to domains of climate change (weather changes, greenhouse gases, anthropogenic drivers and nonspecific climate change) on the right. This diagram includes all reviews, irrespective of directionality, so mechanisms of change can operate in either direction.

Informing action through different research approaches (i.e., research-focused mechanisms of change) was proposed less frequently. Of the reviews critically evaluating research approaches, 20% discussed shifts in research methods (*n* = 172), mostly focused on models (83%, *n* = 143/172), including prediction, econometric, simulation, and systems models. Only 11% of reviews discussed research metrics and measures for either FSNH or climate change (*n* = 96), and <6% discussed changes to data sources and uses (*n* = 49). Figure 8 shows groupings of different mechanisms of change mediating FSNH and climate change factors.

#### Recommendations proposed by authors

The majority of authors included recommendations for future work and action beyond the mechanisms of change they described (91%, *n* = 770). Some reviews did not make specific recommendations (9%). When we classified author recommendations, including future research, policy, and program changes, we found that changes in practices, including production or supply management, behaviors, and systems, were the most commonly recommended (58%, *n* = 449). This could have included strategies to manage heat stress among livestock or types of messaging that influence dietary choices. Changes to research focus, including specific comments on filling gaps and creating new evidence, was the second most common recommendation (38%, *n* = 296), followed by changes to policy and governance (36%, *n* = 278). Common recommendations around filling research gaps included things such as using certain types of models (consequential life cycle analysis) or collecting data to understand contextual factors in interventions or actions. Suggested changes to policy or governance were often broad, such as making financing schemes more accessible, imposing new taxes and tariffs, or supporting certain innovations in production. Changes to technology were recommended for 28% of solution-oriented papers, which often included breeding improvements to seeds or varietals. Changes to knowledge, including decision support and education, changes to research methods and frameworks, and more interdisciplinary and cross-sectoral collaboration were each recommended by ∼20% of papers. Changes in resource access or availability (10%, *n* = 78) and inclusion of equity or changes to equitable approaches were the least mentioned in reports (7%, *n* = 52). These groups of recommendations can be selected as filters in the EGM and papers assessed by the users regarding their specific recommendations.

### Expert consultation

We received 18 responses from the expert consultation, who gave input on the following: *1*) landmark literature on climate change and FSNH, *2*) research/evidence gaps and recommendations, and *3*) promising ways forward, especially for research tools, methods, and metrics. We summarized responses and presented illustrative quotations of these responses (numbered to maintain anonymity). The full analysis of research gaps and areas for future work are provided in Supplemental Results 2–4.

#### Suggested literature

Some of the key literature recommendations were identified in our literature search [[21], [22], [23], [24]]. Our search strategy did not capture certain relevant reviews because they did not mention “review” or “overview” in the title or abstract [5,[25], [26], [27]], did not have an abstract, was not indexed [2,[28], [29], [30]], or were housed in unsearched grey literature databases [[31], [32], [33], [34], [35], [36]]. These reviews were added for data extraction. Some recommended papers did not fit inclusion criteria (e.g., full textbooks, primary analyses, or papers not yet published), so they were not included in the EGM.

#### Research gaps

The expert consultation identified critical research gaps and suggested priorities for advancing understanding of the climate change-FSNH nexus. Broad themes mentioned by many participants included increasing research within postharvest subsectors of food systems. It also included further understanding of climate change impacts, both on food system functions and on the nutrition and health of understudied and marginalized population groups. Many mentioned interactions of climate impacts with socioeconomic and political contexts and cascading risks across systems and sectors. Improving knowledge of food systems vulnerabilities and adaptive capacity was highlighted alongside strong calls for evidence on climate mitigation and adaptation strategies. Several experts noted a need to know about the effects of combining strategies, and the feasibility and translation of strategies for different contexts. They also highlighted a dearth of information on the impacts of these approaches on nutrition and health, including unintended outcomes. They argued a need for new and accessible data, research approaches that address equity and include indigenous knowledge, advancing analytical models that capture complex systems and dynamics, interacting risks and impacts, and increasing the availability of tools that can inform research and policy. Illustrative quotes are highlighted in Box 1.

Box 1 **Illustrative quotes from experts about current research gaps on climate change linked to food systems, nutrition, and nutrition-related health**

“The majority of food retailers believe they can adapt their supply chains very quickly, but we actually do not know the stress limits of this or how it might affect nutrition and health in the future.” (Respondent 1)

“Middle parts of the chain are a huge research gap, such as commodity structures, processing, and transport, as most of us eat food-like substances. These parts of the food chain can be modeled in a much more rigorous way, particularly transportation as a contributor to or mitigator of climate change.” (Respondent 5)

“Most of the literature focuses on fairly simple pathways (e.g., heat stress affecting food production) rather than more complex interactions such as climate change compounding other drivers which affect agriculture, nutrition, and health, or having unanticipated outcomes.” (Respondent 9)

“Research gaps include understanding which strategies are adaptable to various contexts (particularly those most vulnerable to climate change), their impacts on nutrition and health outcomes, and equity outcomes.” (Respondent 4)

“The basic story of migration from the evidence so far is that the poorest and most vulnerable move very little – for them, there will be more climate-poverty traps than they will be climate refugees.” (Respondent 3)

“There is a gap in integrating indigenous and local knowledge into research on climate change adaptation and mitigation (climate-resilient agriculture) and policies. Indigenous communities are particularly vulnerable to the effects of climate change, and also hold important knowledge on climate resilience.” (Respondent 4)

Alt-text: Box 1

#### Expert-identified directions for future research

Suggestions for future directions in research on climate change related to FSNH echoed the gaps respondents identified and the key recommendations made by authors of reports included in the EGM. Broadly, respondents mentioned the need to identify which kinds of data, research methods and approaches would inform food systems action. Some questioned whether macro- or micro-systems focus would be most informative for identifying levers of change or be most useful for decision-makers at various levels. Respondents noted that methods are more evolved to measure the impacts of acute events or disasters but much less evolved for slower (but no less profound) changes such as pest infestation, nonacute flooding, or temperature changes. The inability to capture the spatial-temporal aspects of changing climates, food systems, and health poses challenges to planning effective interventions. They also raised issues of cost modeling and political economy analysis to figure out where the onus for the transformation of environments, food systems, and nutrition should lie. Further integration across disciplines, sectors, and thematic research communities was posed as an important future direction. Illustrative quotations from experts are listed in Box 2.

Box 2 **Illustrative quotes from experts about future directions for research on climate change linked to food systems, nutrition, and nutrition-related health**

Progress “requires collaboration across lots of disciplines and scales.” (Respondent 8)

Even given proper data and perfect models, there will need to be “supporting capacities to use the tools, realize value around tools,” and enable decision making. (Respondent 6)

“There is a big push to collect metrics at various scales and of different dimensions of food systems… We need to… explore how some of those metrics could be projected into the future and related to climate change. There is a need for funders, policy makers and private sector incumbents to come together to… improve data collection and accessibility.” (Respondent 8)

“True validation of… indicators commonly used to proxy for dimensions of climate, planet, food system, health, economics, justice dimensions is still needed.” (Respondent 10)

“Qualitative and quantitative evaluations of climate risk impacts on FSNH among LMICs should also be developed, particularly regarding agriculture adaption strategies.” (Respondent 4)

“Models and tools that can compare impacts and trade-offs from different policies, programs and actions” (would be transformative in the field). (Respondent 2)

“There is a distinct lack of decision tools that are freely accessible, low-input, and do not require extensive data knowledge available to policy makers and government employees. (We need) ways to look at food systems in totality and triggers for performance. We are not currently building tools that are relevant for governments.” (Respondent 5)

Abbreviations: FSNH, food systems, nutrition, and nutrition-related health; LMIC, low- and middle-income country.

Alt-text: Box 2

An overall conclusion from the consultation was that there are 2 different data-related gaps to fill: *1*) models and analyses built by experts that include much more data and types of data and, critically, have better precision and predictive capacity even in the face of changing scenarios; and *2*) effective tools that are straightforward, easy and free to use by nonexperts, that have enough granularity for decision making at a subnational level or above. Several approaches or principles were mentioned as essential to ensure equitable progress on both human and planetary health, especially feminism, bottom-up, participatory approaches and coproduced solutions, and biodiversity and agroecology.

#### Integration with the EGM

Overall, the thematic gaps and the contextual elucidation of gaps and future directions evident in the consultation matched well with the EGM results. Using initial responses from the expert consultation and an iterative approach to re-formulating (and then recoding), the key recommendation categories allowed us to triangulate the EGM results and the responses from the expert consultation across our data sources.

There was a notable overlap between the results of the consultation and the EGM in the lack of reviews on postharvest systems (including processing, packaging, and transport) (mentioned by half of the respondents in the consultation). Food and/or nutrient loss and waste, especially issues of perishability and infrastructure, were commonly mentioned by experts and underrepresented in the EGM. Another example of overlap was the lack of reviews focused on food environments and dietary considerations such as accessibility and affordability. Many expert respondents brought up different aspects of equity and vulnerability, albeit from different perspectives. Research gaps in subdomains, such as vector-borne and infectious disease in the health domain or biodiversity in the agro-environment domain, were also overlapping gaps.

Some gaps mentioned in the expert consultation were, in fact, areas of clustering of research reports in the EGM, for instance, the synthesis of research on the nutrient quality of crops or climate-responsive practices or programs. These are examples of where much literature exists but may not have yet translated into clear, actionable ways forward, especially given that most review types were not systematic reviews or meta-analyses. Experts noted that despite the proliferation of reporting on these topics, the scientific literature falls short of being useful for program and policy design.

## Discussion

We visualize the range, nature, and extent of systematically identified reviews in the past 5 y, analytically linking climate change to FSNH. We analyzed 844 of these reports and mapped them onto an interactive EGM, which can be narrowed through an extensive list of characteristics based on user interest. Although this approach may not capture every primary research theme, the map is supplemented by a stakeholder consultation to comprehensively identify clusters of research interest, or lack thereof, that are instructive for future investments. It may be naïve to think that consensus is possible on some of these intersections – rather, efforts to engage in productive, just dialog, understand bias and influence, and trade-offs and impacts at scale may help prioritize ways forward [37].

### Trends

The largest cluster of research linking climate change to FSNH were studies examining the impacts of weather changes on general crop and ASF production. Reports assessing livestock emission mitigation via biotechnological and animal feed were also prevalent. The mitigation of emissions and the adaptive capacities for soil health, the microbiome, and water systems were notable components of the literature landscape. Much of this literature focused on implementation practices and technologies, described as ways forward in bolstering climate adaptation and mitigation strategies, particularly farm management practices and genetics or breeding. Although there was less literature on pathways to diets and nutrition than in other FSNH domains (e.g., agriculture), food security and diets were more commonly reviewed than nutrition-related health (including underweight, overweight/obesity, and micronutrient deficiencies). These papers were mostly presented as health outcomes from changing weather patterns, although a few diet papers evaluated consumer-driven mitigation of GHGs or impacts of combustion air pollution on respiratory health related to diet-related illnesses. There was virtually no mention of nutrition-related health as a driver of climate change (much less changing nutrition-related health as a climate mitigation strategy), perhaps reflecting that this direction of the relationship may be less intuitive but is not outside the realm of possibility (e.g., increased resources use by those with poor nutrition-related health).

### Gaps: a roadmap for future research

Four main research gaps stand out from our EGM analysis and stakeholder consultation.

First, comparatively few reports addressed whole systems or the “middle” of food systems, i.e., postharvest and before consumption and nutrition outcomes. For instance, although many reports broadly examined food systems, there were few reviews on how climate change might influence food prices and expenditure, food safety, food processing, food environments, or supply chains. Despite prevalent global discussions about transforming supply chains, food environments, and climate-smart consumer choices, there were few reports in the EGM representing this topic. Because most people now eat foods that have been stored, transported, processed, or transformed in some way or other, which heavily contributes to GHG emissions, this may be a key strategy for climate mitigation [38].

Second, few papers critically assessed pathways from climate change to nutrition-related health. Some obvious pathways, such as climate-induced increases in morbidity and mortality in populations having high obesity and cardiovascular disease, were represented to some degree (and explicitly part of our search strategy). Other nutrition-related health aspects were virtually absent; for instance, there was some mention of disease vectors[12,39], but they were not always explicitly linked to micronutrient deficiencies (e.g., malaria and dengue). Because global warming is making malaria and dengue more prevalent in areas with high rates or risk of iron deficiencies and anemia, these populations will experience a greater burden of corollary health consequences [40]. The lack of reviews on these topics could also be because the search strategy was less explicit (but not exclusive) for these intersections. There are many other instances where the impact of climate change on health via nutrition is unclear (e.g., destruction of homes and livelihoods in populations already nutritionally at-risk), which will deepen and widen extant disparities. These are just a few examples of the multiple complex interactions and feedback cycles that will undoubtedly play a burgeoning role in our global health outlook going forward. Thus, knowing more about these scenarios could help us offset harm and apportion resources more effectively.

Third, despite advances in climate modeling, it is still unclear which methods are optimal in informing climate adaptation and mitigation strategies for different food systems and their component parts. For instance, methods that can identify the population-attributable benefits and risks of different approaches and actions, and those that will be less effective overall, are sorely needed.

As part of this point, few analytical methods can evaluate trade-offs and complex feedback loops, which will also be useful in identifying intervention opportunities with the greatest impact. Many reviews were very broad and contributed few solutions to well-established problems. Characterizing the nature and extent of problems at the intersection of climate change and FSNH is a first step toward problem-solving but may have limited utility in decision making by producers, consumers, or governments. This is especially true in the context of difficult and complex trade-offs that should be considered when planning for improved resilience in food systems and nutrition. For instance, many reviews framed all types of weather events as risk factors. Although this might be true in some cases, climate modelers from the consultation assert that there will actually be mixed impacts on agriculture, whereby some aspects of climate change will benefit certain parts of food systems in certain regions, climates, or contexts but pose harm or challenges to others [41]. Almost all agree that the uncertainty thresholds are too wide – that existing models do not account for myriad influences and the complexity that will unfold. The EGM indicates little synthesized research on analytical modeling, metrics, or approaches informing dynamic climate adaptation strategies. Of course, no model can account for every dynamic, but there are many disciplinary “blind spots” that can be elucidated to know what is truly possible or available methodologically (e.g., through using the EGM). Additionally, our approach overall may have limitations. For instance, systematic reviews are considered the gold standard in public health disciplines but not in environmental science, and thus, “state of the art” evidence might not be represented as fully for some disciplines by searching for reviews alone.

The common follow-on to arguments about methodological limitations is to propose more tools and models that can handle complexity and narrow confidence estimates, allowing more accuracy and precision in predicting outcomes. This would include, for instance, the mitigating potential of combining reduced ASF consumption with improved soil practices or the adaptive potential for more diverse seed stock combined with better conditions for agricultural workers who suffer heat stress. There is certainly a place for methods that can tolerate more diverse types of information and improve data quality. For instance, many types of climate data are already collected as spatial time series (from weather stations or satellites), but FSNH data types are not collected as frequently among representative populations and are rarely publicly available at high spatial resolutions (e.g., subnational administrative boundaries or Global Positioning System coordinates), thus limiting our ability to link climate change to food systems and health status. Large amounts of data that could answer crucial questions are collected and owned by the private sector. However, as extensively argued [[42], [43], [44]], collaborating with the private sector is contentious, especially due to conflicts of interest, incentive structures, enacting effective policy, and the lack of political economy analysis from which to learn. Data gaps are nonetheless notable, especially as they relate to our ability to dynamically respond to changing situations.

An additional gap in methodology is the lack of reviews in the EGM that are context specific. The EGM is dominated by papers that are global in scope, with only a third synthesizing regional, country, and subnational research. Furthermore, because most reviews were subject reviews (only 10% were systematic reviews), these papers represent thematic interest but often lacked crucial rigor on synthesis required for decision making. The goal of any research in this area should be to inform decision-makers and generate impact. However, if most of the research generated is vague or so aggregated to a global scale that much of the nuance is lost (whether it be around local trade-offs or specific dynamics, etc.), this will be of finite use to policy- and decision-makers at national and local levels. Even the most considered, inclusive quantitative global models that exist currently are rarely held accountable for poor predictive capacity or limited utility. Although global models will always have their place in international agenda-setting, there is much scope to improve research available to those making decisions and policies for their specific communities.

Indeed, some of the most promising methodological approaches that can fill these gaps may go beyond or complement global statistical models. Notable in the EGM and our consultation was a lack of application of political economy analysis, scenario-based research, advanced life cycle, and input/output-related methods, qualitative and ethnographic analysis, and others. Some of these methods could address both the need to holistically assess systems (rather than silos) and optimize investment to have the most impact, as well as include contextual factors and equity.

Fourth, collaboration will be key in producing research, assessing evidence, and translating evidence into action. There are many examples of stagnant policy and lack of political will or behavior change, even with well-founded information at hand. From both the EGM and the consultation, it is clear that research gaps are compounded by serious barriers to the uptake of information or practice, from climate-smart agriculture in sub-Saharan Africa to sustainable diets in high-income contexts to trade policy changes. Plenty of evidence delineates the problems surrounding climate change and FSNH and even what needs to change, but how we do so locally and at scale is still unclear. All experts agreed that interdisciplinary and intersectoral collaborations will be crucial in furthering such research.

Further to this point, some of the more explanatory syntheses point to action being inhibited by inequity. For instance, several reviews argued that barriers to the uptake of agro-environmental practices were due to inequities in gender, social capital, socioeconomic status, and place of residence. Overall, though, there was a striking lack of literature critically engaging with equity when linking climate change with FSNH. Even from the reviews that iterated issues of access, affordability, risk, and vulnerability, only 6% of reviews overall recommended changes to equity and equitable practices. This raises fundamental questions about whether and to what degree the research community empirically engages with the premise that equity is not only a desired outcome for food security and nutrition but a strategy in itself for realizing effective actions that synergistically improve diets and planetary health for all [45]. It also points to questions of who will pay for the transformation of food systems, as well as what will catalyze political action. Equity considerations will be paramount in any of these analyses and approaches if we are to ensure that the onus for change is shouldered realistically and without inducing more vulnerability among those most at-risk.

In conclusion, this map – a wide landscape of research linking climate change to FSNH – is the first of its kind. We imagine that despite its descriptive nature, it is a useful repository of the most prominent research on these linkages to date and a powerful visualization of research that is missing. Whether these gaps are worthy of further investment will depend on the following: *1*) the interdisciplinary expertise and focus of those working at certain intersections, *2*) an analysis of how these gaps align or don’t align to impact frameworks and pressing needs, and *3*) whether filling these gaps will spur action or simply add to the noise.

The key gaps highlighted by both EGM and consultation were 4-fold: *1*) research focusing on whole systems and postharvest food systems, including overlooked aspects of food processing, packaging, distribution, and waste; *2*) research evaluating how climate change affects nutrition-related health, such as heat stress and disease vectors that overlap with nutritional factors, food-related livelihoods and displacement among nutritionally vulnerable populations; *3*) promising methods (and additional data required) that can i) identify inflection points for intervention, ii) incorporate complex dynamics and feedback loops as well as characterize trade-offs, and iii) be available, understood and applied in context-specific, localized ways for decision making; and iv) both producing and translating evidence to action will be enabled by interdisciplinary and cross-sectoral collaborations, especially those that inherently consider equity, coproduction, and fairness. Specifically, actively promoting communities most impacted by climate change (especially those already marginalized) as participants in research and decision making will bolster the successful translation of research into action. In turn, this will generate significant global benefits because these population-attributable effects will be the greatest.

This research mapping and stakeholder engagement is a critical overview of a rapidly evolving intersection of research fields with serious implications for coping mechanisms, potential solutions, and real-world impacts. Identifying the most pressing research needs is a matter of protecting planetary and human health against the clock of climate change.

## Acknowledgments

We thank the Innovative Methods and Metrics for Agriculture and Nutrition Actions team for their ideas, logistical and dissemination support: Ore Kolade, Chau Yo, Ana Sousa, Babatunde Makanjuola, and Indira Bose, especially Joe Yates for contributions to the introduction, as well as Claire Dooley, Zip Walton, and Emily Fivian for their thoughts on preliminary reporting structures. We wholeheartedly thank each expert consultant for their informative and thoughtful responses to our questions: Ramya Ambikapathi, Rachel Bezner-Kerr, Megan Deeney, Jess Fanzo, Bridget Fenn, Upasona Ghosh, Rosemary Green, Robert Hughes, Lindsay Jaacks, Jonas Jaegermeyr, Nathalie Lambrecht, Tafadzwa Mabhaudhi, Will Masters, Bhavani Shankar, Christopher Trisos, Roosmarijn Verstraeten, Patrick Webb, and Keith Wiebe.

## Author contributions

The authors’ responsibilities were as follows – MD, CO: first responded to a request to produce this work for the Innovative Methods and Metrics for Agriculture and Nutrition Actions (IMMANA) funders. MD, CO, TMS: further revised the scope and approach, with support from SK, the IMMANA team, Foreign Commonwealth Development Office, and Bill and Melinda Gates Foundation; CO: built the search strategy with support from TS, and both designed the screening and coding materials; TMS, CO: led a team of PD, KB, RJ, SM to screen and code all records; CO: assisted by TMS, revised and reviewed coding and conducted the quantitative analysis; TMS: assisted by CO, conducted the qualitative analysis, drafted the manuscript, maps, and figures, with additional assistance from MD; and all authors: read and approved the final manuscript.

### Conflict of interest

The authors report no conflicts of interest.

## Funding

This work was supported by the Innovative Methods and Metrics for Agriculture and Nutrition Actions program, funded by Foreign Commonwelath Development Office and the Bill and Melinda Gates Foundation. These funders proposed this project and suggested the initial scope but had no role in the overall design, implementation, analysis, or interpretation of the data.

## Data availability

All data generated or analyzed during this study are included in the HTML Evidence and Gap Map and accompanying paper (and its supplementary information files). The full database (including initial search results and screening codes) can be accessed upon request, to the author.
