# Supplementary material for: Intersections of Climate Change with Food Systems, Nutrition, and Health: An Overview and Evidence Map
Source: Adv Nutr. 2024 Jul 15;15(9):100274. doi: 10.1016/j.advnut.2024.100274 (PMC11382032; doi:10.1016/j.advnut.2024.100274)
Supplement: Multimedia component 1 [file mmc1.docx]

# Supplement

### Supplemental Methods 1: Methodological strengths and limitations of our approach

We searched three indexing databases (Web of Science, Scopus and Medline) and three grey literature sites (Global Index Medicus, the World Bank, 3IE and CGIAR databases. We chose to include published reports that discussed existing literature, i.e. all kinds of reviews.  This included subject matter reviews, non-systematic literature reviews, scoping and systematic reviews, meta-analyses, policy reviews and overviews (i.e. reviews of reviews).

Strengths:

- Includes a wide breadth of thinking and discussion on this large topical intersection.
- Includes thought pieces that draw on evidence but may go beyond a narrow methodological approach to bring in wider themes/points.
- Is more able to capture interdisciplinary reporting norms, e.g. in STEM sciences, literature reviews follow different conventions than in public health.
- The EGM can be limited or filtered by type of review; formats viewed as less rigorous or relevant can be excluded.

Limitations:

- Included reviews do not always follow the same methodology, which may limit the ability to compare key findings.
- While subject matter reviews may be organized to support a certain interest, they may not systematically represent all the relevant literature on the topic, or present a clear focus, methodology, or approach to assessing the quality of included evidence.
- Due to the varied nature of included reviews, it was not within this projects’ scope to investigate the quality of the underlying literature presented, nor the strength of the conclusions reached.

### Supplemental Methods 2: Example search string from Web of Science database. Where blue indicates climate change terms; purple indicates food systems terms; yellow indicates nutrition & health terms; and Green indicates agriculture terms

*(TI=****( (****climate OR "uncertain climate" OR "climate variability" OR "global warming" OR "greenhouse gas*" OR GHG OR emission* OR environment* OR “Net zero” OR “net-zero” OR "extreme weather" OR flood* OR drought* OR hurricane* OR typhoon* OR (ris* adh2 ("sea-levels" OR "water levels")) OR salinity OR acidification OR "rising temperature*" OR "water temperature*" OR "rainfall patterns" OR (heat adh2 (stress OR extreme)) OR (storm* adhd (extreme OR tropical))* ***)*** ***AND (*** *(diet* OR nutrient* OR nutrition* OR (health adhd nutrition*) OR malnutrition OR malnourish* OR underweight OR "under weight" OR “under nourish" OR undernourish* OR undernutrition OR kwashiorkor OR marasmus OR ((body OR bodily) adh2 (thin OR thinness)) OR obes* OR overweight OR "over weight" OR "body measur*" OR BMI OR "body mass index" OR diabet* OR "hyper-tension" OR hypertension OR "cardio-vascular" OR cardiovascular OR micronutrient* OR ((iron or iodine or vitamin* or zinc) adhd deficien*))* ***OR*** *(food* adh2 (system* OR produc* OR safety OR process* OR loss* OR stor* OR policy OR policies OR security OR insecurity OR consum* OR expend* OR environment OR legislat* OR market* OR price OR prices))* ***OR*** *(agri* OR agro* OR farm* OR horticulture OR livestock OR husbandry OR aquaculture OR "fish farming" OR ((food* OR crop*) adh2 (produc* OR grow* OR cultivat* OR rais* OR harvest* OR loss* OR stor*))))* ***) )*** ***AND*** *(TI=(overview* OR review*) OR AB=(overview* OR review*))* ***AND*** *PY=(2018-2023)*

### Supplemental Methods 3: Inclusion and exclusion criterea for phase 1 and 2 of the review screening process. Text formatting styles were used to emphasis key points of inclusion (**bold**) and exclusion (underline) criteria.

| ***INCLUSION FACTORS*** | ***EXCLUSION FACTORS*** |  |
| --- | --- | --- |
| ***Publication Year*** | | |
| Published between January 2018 and February 2023 | Studies published prior to January 1 2018 |  |
| ***Literature Type*** | | |
| Published synthesis research (peer-reviewed), and peer-reviewed book chapters | Unsuitable literature to exclude:   - Conference abstracts - Theses - Studies reported in non-English languages - Retracted papers - Errata/Corrections - Protocols - No abstract (but check google first) - Whole books |  |
| ***Type of Review (Study design)*** | | |
| Synthesis studies to include:   - Cochrane Reviews - Cochrane Overviews of Reviews - Systematic Reviews - Overview Reviews - Umbrella Reviews - Reviews of Reviews - Case study reviews - Qualitative Reviews - Narrative Reviews - Policy Reviews - Mini Review - Meta-analyses | Non-synthesis studies to exclude:   - Single experimental or non-experimental studies - Case studies - Literature reviews with the purpose of providing foundation for a primary analysis |  |
| ***Population*** | | |
| Populations to include:   - **General populations** of any age, gender or global geography - Review focussed on **human nutrition/health outcomes** (i.e. diets, nutrient intake, food security) - Reviews focussed on **non-human agriculture/food system outcomes** (i.e. crop productivity/yield; poultry productivity) | Non-generalizable populations to exclude:   - Studies examining nutrition/health outcomes of non-human populations (i.e. diets of pig/cattle/bacteria/fish) - Specific or niche human populations with uncommon nutritional patterns, (i.e. elite athletes; those suffering eating disorders, etc.) |  |
| ***Exposures/Outcomes*** | | |
| Climate Change (relevant):   - Any review examining **CC-explicit** exposures/outcomes (i.e. "Climate Change/ Variability/ Uncertainty", "Global Warming", "Greenhouse Gases ", "Carbon/Nitrogen/Methane Emissions" or "Extreme Weather/Environments" or “Anthropocene")   - **GHGs**: Carbon dioxide; Methane; Nitrous Oxide; Sulfur; Nitrogen Trifluoride; Hexafluoride; Chlorofluorocarbon-12; Hydrofluorocarbon-23.   - **Extreme Weather;** Rising Temperatures; Rising sea levels; Changing precipitation/ rainfall patterns; Extreme flooding; Extreme droughts; Extreme storms (hurricanes, typhoons) - Any review examining **CC-implicit exposures/outcomes** (i.e. “environmental impact/ sustainability/ hazards" or "eco-friendly/organic" or "LCA/circulatory") - Any review examining **CC-implicit phenomena** (i.e. air pollution, salinity, floods, droughts, meat-alternatives, etc.) | Climate Change (not relevant):   - Exclude reviews examining unrelated mitigation/ adaption outcome/exposure (i.e. dietary mitigation for poultry production). - Exclude reviews examining unrelated emissions outcome/exposure (i.e. fMRI/ tomography emissions) - Exclude studies examining unrelated environment phenomena (i.e. local/built environments) - Exclude reviews of studies examining unrelated climate outcomes/exposures (i.e. not climate change explicitly, environmental changes not linked to climate change) - Exclude studies examining individual/isolated weather events (i.e. Hurricane Katrina) |  |
| Food Systems, Nutrition & Health (relevant):   - Reviews can be focused on **any part of the food system** (i.e. single or specific crops, biofuels, livestock, diets, pests, food waste, fertilisers, etc.) - Reviews examining **nutrition-related health outcomes** (i.e. diabetes/ cardiovascular/ chronic diseases) IF they meet other inclusion criteria - Reviews can examine **plant,** **aquaculture and livestock genetics**, but must meet other inclusion criteria | Food Systems, Nutrition & Health (not relevant):   - Exclude studies related to ecology, forestry, or minerals WITHOUT explicit links to food systems or nutrition (if they are linked in abstract, then include study) - Exclude studies with non-nutrition health exposures/outcomes (i.e. air pollution & cardiovascular health) |  |
| ***Analytical Link*** | |  |
| Any review of studies explicitly examining the empirically relationship between: [Climate Change / Global Warming / Greenhouse Gas / Emissions / Extreme Weather / Anthropocene] **AND** [Agriculture / Nutrition/ Nutrition-related Health / Food Systems/ Aquaculture / Livestock]  Reviews examining the analytical **link between CC-FSNH in either direction** (CC🡪 FSNH *OR* CC 🡨 FSNH) | Exclude if the review does not explicitly link to Climate Change OR ANH/Food systems(i.e. as a clear outcome/exposure)  Exclude reviews on both ANH/food systems and climate change as separate subjects should be excluded. |  |

### Supplemental Methods 4: Data coding

#### A. Categorization of data extraction characteristics

We first created the framework (rows and columns of the map) deductively using established frameworks (described further below). Because of thematic overlap in most frameworks, we also refined and organised the sub-domains of the rows and columns iteratively (although still in reference to the frameworks) through testing an initial set of records. Other characteristics of studies were decided a priori, such as setting and population of interest. Further categories, such as mechanisms of change and author recommendations were created inductively as a method of ‘horizon scanning’ (and because there are no established frameworks for these). We thought that these especially would be highly valuable for various types of users of the map (including policy-makers and donors). For these categories, initial codes for these sections were identified from a sample selection and then supplemented by reviewer knowledge of included papers and the expert consultation. After the first round of coding, themes were finalised and we reviewed and re-coded all coded records according to the new structure.

The Climate Change domain (presented as the rows in the EGM) was broadly divided into Emissions (i.e. Greenhouse Gases) and weather changes (e.g. precipitation, temperature, extreme events). Although other anthropogenic drivers of climate change (e.g. acidification, salinization, pollution) were not included in the search strategy, they were coded as themes if reports fit the overarching inclusion criteria.

FSNH domains were decided through both grounded and iterative methodologies. We used existing Agriculture, Nutrition and Health (ANH) and Food Systems frameworks, such as the High-Level Panel of Experts (HLPE) reports^[[1]](#footnote-1)^, to determine domains and subdomains. However, creating a ‘flat’ or linear organization of domains is subjective, as there are many overlaps in frameworks (e.g. policy aspects being external drivers, cross-cutting and listed in several ‘nodes’ of the HLPE; food quality being part of supply chains, food environments and diets; or food prices and expenditure cross-represented in food security pillars, food environments, consumer behaviour and diets). Therefore, we organised this section iteratively as it reflected the literature. Most reports covering these interrelated themes were coded in several domains and subdomains, and thus can be easily identified regardless of the prevailing approach of the reader.

Reviews were grouped into various types, including subject matter reviews with no stated methodology, literature reviews that presented their approach to literature but did not follow systematic reporting guidelines, systematic reviews (following PRISMA or ROSES), overview of reviews, (defined by Cochrane as reviews only including other reviews^[[2]](#footnote-2)^), policy or governance reviews, and meta-analyses or other pooled analyses where results are quantitatively combined to create an overarching estimate of effect.

We characterised directionality as the overarching hypothesis motivating a report’s research question or aim. Included reviews were coded as having a general hypothesis of climate change driving FSNH, FSNH driving climate change or a bi-directional relationship (focus on both directions). For information on populations, many reviews focused on elements of the FSNH pathway that did not directly involve a human population (e.g. animal feed or plant nutrient cycles): these were coded as not applicable. We used the Cochrane PROGRESS+ framework^[[3]](#footnote-3)^ to identify papers that critically discussed various aspects of equity. Reviews not engaging with any dimension of equity were coded as none.

We summarized any factors identified by authors as modifying, mediating or transforming the climate change-FSNH relationship, which we termed ‘Mechanisms of Change’. Mechanisms of change were grouped as: policy; technology; Knowledge, Attitudes and Practices (KAP; see Box 1); research methods, metrics and data sources. Key recommendations stated by authors were also grouped, with further attention given to collaboration, resources, access, and equity, as these were also themes mentioned in the consultation. Some authors did not offer specific key recommendations, so these reports were broadly grouped into ‘none’. Studies could cover more than one aspect of different domains and various characteristics. Therefore proportions presented are not additive; rather they reflect the prominent components of each report and can be selected based on user interest. Section B provides an exhaustive list of the coding structure and definitions.

| *Box 1: What are Knowledge, Attitudes, Practices (KAP) in this EGM?*  KAP is a methodological approach to assessing people's understanding (knowledge), beliefs and perceptions (attitude), and actions (practice) related to a specific topic or issue (45). It is commonly employed in public health, social sciences, and market research to gather valuable insights about various aspects of human behaviour and decision-making. We have adapted KAP concepts for this review in the following ways:   - Knowledge: refers to the information and awareness individuals or groups have (or don’t have) about CC-FSNH (i.e. collaborative knowledge, indigenous knowledge, decision support and services, education) - Attitudes: encompasses to people's opinions, beliefs, and attitudes towards CC-FSNH (i.e. CC perceptions, consumption behaviours or preferences, attitudes towards adaptation) - Practices: relate to performed behaviours and actions of individuals, organisations or systems, including the management practices that are deployed at different parts of the food system (i.e. GHG sequestration, climate-smart practices or other agro-environmental/ crop/ animal/ fishery/ water/ business/ institutional practices) |
| --- |

#### B. Coding structure for data extraction with relevant definitions

| **THEMATIC AREA** | **SUB-HEADINGS** | **DEFINITIONS** |
| --- | --- | --- |
| **Type of Review (Study Design)** | Subject Review (i.e. non-methodological) | Non-methodological subject matter or content review |
|  | Literature Review (i.e. non-systematic) | A review of literature with some stated methodology, but not following systematic review guidelines. |
|  | Systematic Review (i.e. PRISMA or ROSES) | A review of literature that follows standardised guidelines, e.g. PRISMA or ROSES |
|  | Scoping Review (i.e. research landscapes) | Review of broad thematic literature and evidence gaps |
|  | Overview Review (i.e. review of reviews) | Review of other reviews, usually following Cochrane definition^[[4]](#footnote-4)^ (i.e. using explicit and systematic methods to search for and identify multiple systematic reviews on a similar topic for the purpose of extracting and analysing their results across important outcomes). |
|  | Policy, Organisational Review | Review of policies and/or policy documents, including organisational documentation. |
|  | Meta, Pooled Analysis | Statistical combining of the results of multiple studies. |
|  | Bibliometric Analysis | Analysis based on citation counts and patterns, usually includes a bibliometric network diagram. |
| **Directionality (domain related)** | CC drives FSNH outcomes | The hypothesised direction of studied relationship based on the research or aims of the paper. |
|  | FSNH drives CC outcomes |  |
|  | Bi-directional (examines both) |  |
| **Domain: Climate Change** | *CC Domain: Non-specific Climate Change* | We define climate change as “large-scale, long-term shift in the planet's weather patterns and average temperatures resulting from anthropogenic greenhouse gas emissions”.  Thus, we included any paper examining explicit CC exposures/outcomes as their research focus, particularly regarding GHGs and weather changes. Codes were selected if topic was *either* explicitly linked OR implied in its research focus AND critically analysed in the methods/results/discussion.  The “non-specific” codes were used for papers specifically referring to an area without any further detail OR subject review papers that may mention multiple aspects, but at no point define the remit of interest (i.e. about climate change, and mentions emissions or weather patterns in passing).  We coded any *additional* anthropogenic drivers that were explicitly linked to the research focus AND critically analysed in the methods/results/discussion. These are considered additional because they were not explicitly included in our search string.  Further code clarifications:   - AD Acidification & Salinization: apply to both soil and water bodies - AD Environmental Pollutants: includes toxic chemicals, pollution & plastics |
|  | *CC Domain: Greenhouse Gases (GHG)* |  |
|  | GHG: Non-specific GHGs |  |
|  | GHG: Carbon |  |
|  | GHG: Nitrogen |  |
|  | GHG: Methane |  |
|  | GHG: Other (e.g., CFC, Triflouride, Sulfer dioxide) |  |
|  | GHG: Ammonia |  |
|  | GHG: Combustion Air Pollution |  |
|  | *CC Domain: Weather Changes (W)* |  |
|  | W: Non-specific Weather |  |
|  | W: Ambient Temperature |  |
|  | W: Rising Sea Levels |  |
|  | W: Precipitation Patterns |  |
|  | W: Flooding |  |
|  | W: Droughts |  |
|  | W: Storms |  |
|  | W: Climatic Extremes (i.e. events, disasters, shocks) |  |
|  | W: Climatic Stress (i.e. heat & water stress) |  |
|  | W: Abiotic Stress |  |
|  | *CC Domain: Other Anthropogenic Drivers (AD)* |  |
|  | AD: Acidification |  |
|  | AD: Salinization |  |
|  | AD: Environmental Degradation |  |
|  | AD: Environmental Pollutants |  |
|  | AD: Resource Scarcity, Population Growth |  |
|  | AD: Energy |  |
| **Domain: Food Systems, Nutrition & Health (FSNH)** | *FSNH Domain: Agro-environmental (ENV)* | We defined FSNH as the entire range of actors and their interlinked value-adding activities involved in the production, aggregation, processing, distribution, consumption, and disposal (loss or waste) of food products that originate from agriculture (incl. livestock forestry, fisheries) and any subsequent nutrition and nutrition-related health outcomes. This is adapted from the EU commission definition of food systems^[[5]](#footnote-5)^.  Thus, we included any papers examining explicit FSNH exposures/outcomes as their research focus, including agro-environmental, food systems (pre- and post-farmgate), food security, nutrition and nutrition related health.  Codes along this continuum were selected if topic was either explicitly linked to or implied in its research focus AND critically analysed in the methods/results/discussion.  The “non-specific” codes were used for papers specifically referring to an area without any further detail OR subject review papers that may mention multiple aspects, but at no point define the remit of interest  We coded any *additional* nutrition-related health or agro-environmental topics that were explicitly linked to or implied in the research focus AND critically analysed in the methods & discussion sections. These are considered additional because they were not explicitly included in our search string.  Further code clarifications:   - ENV Water Systems: relates to irrigation, water use or management - ENV Bacteria, fungi, microbes: relates to uses or impacts of microbial processes that are not explicitly considered detrimental to organisms. - ENV Pests, diseases, pathogens: relates to uses or impacts of pests and microbial processes that are explicitly considered detrimental to organisms. |
|  | ENV: Soil |  |
|  | ENV: Plant Nutrients |  |
|  | ENV: Bacteria, Fungi, Microbe |  |
|  | ENV: Pests, Diseases, Pathogens |  |
|  | ENV: Weeds |  |
|  | ENV: Land Use |  |
|  | ENV: Biodiversity, Agroecology, Agroforestry |  |
|  | ENV: Water Systems |  |
|  | *FSNH Domain: Primary Food Production* |  |
|  | Non-specific Primary Food Production |  |
|  | Crop Production (CP) |  |
|  | CP: Non-specific |  |
|  | CP: Staple grains |  |
|  | CP: Roots, Tubers |  |
|  | CP: Legumes, Pulses |  |
|  | CP: Vegetables |  |
|  | CP: Fruits |  |
|  | CP: Nuts, Seeds |  |
|  | CP: Herbs, Teas, Coffee, Cacao |  |
|  | CP: Animal Feed Crops |  |
|  | Animal Source Foods (ASF) |  |
|  | ASF: Non-specific |  |
|  | ASF: Ruminant Livestock |  |
|  | ASF: Cattle |  |
|  | ASF: Pigs |  |
|  | ASF: Goats, Sheep |  |
|  | ASF: Poultry |  |
|  | ASF: Dairy |  |
|  | ASF: Rabbits |  |
|  | ASF: Bees, Edible Insects |  |
|  | Aquaculture: Fish, Seafood, Seaweed |  |
|  | Indigenous, Orphan Foods, NUS |  |
|  | *FSNH Domain: Post-harvest Systems* |  |
|  | Non-specific Post-harvest Systems |  |
|  | Food System Policy |  |
|  | Food System Economics |  |
|  | Food System Waste, Loss |  |
|  | Food Supply Chains, Transport |  |
|  | Food Packaging, Labelling |  |
|  | Food Processing |  |
|  | Food Safety |  |
|  | Food Quality, Nutritional Value |  |
|  | Food Prices, Expenditure |  |
|  | Food Environments |  |
|  | *FSNH Domain: Food Security & Diets* |  |
|  | Food Security |  |
|  | Diets |  |
|  | Infant & Child Feeding (IYCF) |  |
|  | *FSNH Domain: Nutrition-related Health* |  |
|  | Malnutrition (MAL) |  |
|  | MAL: Non-specific |  |
|  | MAL: Micronutrients |  |
|  | MAL: Infant Growth (i.e. PTB, SGA, wasting, stunting) |  |
|  | MAL: Undernutrition |  |
|  | MAL: Overnutrition |  |
|  | Non-Communicable Diseases (NCD) |  |
|  | NCD: Diabetes |  |
|  | NCD: Hypertension |  |
|  | NCD: Cardiovascular Health & Disease |  |
|  | NCD: Kidney & Heat-related Illness |  |
|  | NCD: Respiratory Health |  |
|  | NCD: Cancer |  |
|  | NCD: Toxins, Allergies, Immunity |  |
|  | NCD: Accidents, Injury |  |
|  | NCD: Mental Health |  |
|  | Communicable Diseases (CD) |  |
|  | CD: Infectious & Vector borne Diseases |  |
|  | CD: COVID-19 |  |
|  | General Health (i.e. mortality, disability) |  |
| **Strategy (mechanism related)** | Adaptation | Papers examining mechanisms of change along the CC-FSNH pathways are normally related to adaptation or mitigation strategies or both. Many papers critically examining strategies without explicitly linking them and were thus coded as strategies. The “None” code was for papers that did not critically discus any strategy. |
|  | Mitigation |  |
|  | Both |  |
|  | None |  |
| **Mechanism of Change (MoC)** | *MoC: None* | We define mechanisms of change (MoC) as any factors identified by authors as modifying, mediating, or transforming the climate change-FSNH relationship.  We used the following categories of mechanisms: Policy; Technology; Knowledge, Attitudes & Practices (KAP); Methods; Metrics; and Data. The last 3 categories were considered distinct research mechanisms of change. Papers that did NOT explicitly examine a mechanism of change were coded as NA.  Codes were selected if topic was explicitly linked to the research focus AND critically analysed in the methods/results/discussion.  Some MoC codes overlap with CC-FSNH codes. The MoC codes are only selected when the component changes the CC-FSNH pathway – the respective CC-FSNH codes will not be used in this case (e.g. Metal Oxide Nanoparticles Mediate Growth Regulation and Physiology of Crop Plants under Drought – this is coded under CC: drought; FSNH: Crops; MoC: Nanotech & bacteria).  Further code clarifications:   - Biotech Fertilizers & Pesticides: toxicity is coded using env pollutants in climate change domain. - P: Agro-environmental practice includes organic, integrated farming, tillage, agroforestry |
|  | *MoC: Policy, Governance* |  |
|  | *MoC: Technology* |  |
|  | *Non-specific Technology* |  |
|  | *Biotechnology (BIO)* |  |
|  | BIO: Non-specific |  |
|  | BIO: Genetics, GMO's |  |
|  | BIO: Bio-fortification |  |
|  | BIO: Nanotechnology |  |
|  | BIO: Microbiomes |  |
|  | BIO: Bio-stimulants, Hormones |  |
|  | BIO: Fertilizers, Pesticides |  |
|  | BIO: Animal Feed, Diets, Supplements (e.g. tannins) |  |
|  | BIO: Biochar |  |
|  | BIO: Biofuel |  |
|  | BIO: Food Processing |  |
|  | BIO: Bio-degradable Packaging |  |
|  | *Agriculture Technology (i.e. controlled env, precision ag, water tech)* |  |
|  | *Information Technology (i.e. internet, mobiles, GIS)* |  |
|  | *Sensor Technology (i.e. satellite data, data collection devices)* |  |
|  | *Other Technology* |  |
|  | *MoC: Knowledge Attitudes Practices (KAP)* |  |
|  | *Knowledge (K)* |  |
|  | K: Collaborative Knowledge, Social Capital |  |
|  | K: Indigenous & Local Knowledge (ILK) |  |
|  | K: Decision Support, Services |  |
|  | K: Education, Training |  |
|  | *Attitudes (A)* |  |
|  | A: Attitudes, Perceptions, Beliefs |  |
|  | *Practices (P)* |  |
|  | P: GHG Fixation, Sequestration |  |
|  | P: Climate-smart Practices (i.e. CSA, CA) |  |
|  | P: Agro-environmental practices |  |
|  | P: Crop Practices |  |
|  | P: Animal Practices |  |
|  | P: Fishery Practices |  |
|  | P: Water Practices |  |
|  | P: Consumer Practices (i.e. behaviour) |  |
|  | P: Producer Practices (i.e. behaviour) |  |
|  | P: Business & Financial Practices |  |
|  | P: Institutional Practices |  |
|  | *MoC: Methods* |  |
|  | Methods: Frameworks (i.e. approaches, theories) |  |
|  | Methods: Models (MM) |  |
|  | MM: Non-specific Models |  |
|  | MM: Prediction Models |  |
|  | MM: Econometric Models |  |
|  | MM: Simulation Models (i.e. scenarios, agent base models) |  |
|  | MM: Systems Models (i.e. GIS, circular economies) |  |
|  | MM: Life Cycle Assessment (LCA) |  |
|  | MM: Impact/Risk Assessments |  |
|  | MM: Other Mathematical Models |  |
|  | Methods: Other approaches |  |
|  | *MoC: Metrics & Measures* |  |
|  | *MoC: Data Sources & Use* |  |
| **Equity** | *Critically Evaluated: Yes* | Papers were checked for whether they critically evaluated equity (yes/no). Those that did assess equity were categorised according to the Cochrane PROGRESS+ groups.  Evaluation of equity could not involve empirical assessments because most of our papers were not methodological reviews. Equity was thus coded if it was discussed in enough detail for us to categorise it using the PROGRESS+ framework. For instance, mentions of women being poor with no further elaboration did not count, but discussions of women not having access to resources which hindered their ability to adapt to climate change would count. |
|  | Yes: Place of Residence |  |
|  | Yes: Race, Ethnicity, Culture, Language |  |
|  | Yes: Occupation |  |
|  | Yes: Gender, Sex |  |
|  | Yes: Religion |  |
|  | Yes: Education |  |
|  | Yes: Socioeconomic Status |  |
|  | Yes: Social Capital |  |
|  | Yes: Plus |  |
|  | *Critically Evaluated: No* |  |
| **Key Recommendations** | *None* | Papers were check for key recommendations, which broadly fell into categories of “None” (i.e. problem-oriented papers) and “Recommendations” (i.e. solution-oriented papers). Recommendation codes were selected if the suggestion was explicitly provided in the results/discussion/conclusion. Codes are not mutually exclusive: papers can cover multiple recommendations.  Further code clarifications:   - Changes to technology includes changes to innovation itself – not just technological uses. - Changes to Research: only selected if paper provides VERY specific changes to research agendas. |
|  | *Recommendations* |  |
|  | R: Changes to Policy |  |
|  | R: Changes to Technology |  |
|  | R: Changes to Knowledge: decision support, education |  |
|  | R: Changes to Attitudes: perceptions, beliefs |  |
|  | R: Changes to Practice: behaviours, management, systems |  |
|  | R: Changes to Research: focus, gaps, evidence |  |
|  | R: Changes to Research: methods, frameworks, models |  |
|  | R: Changes to Research: metrics, measures |  |
|  | R: Changes to Data: types, frequency, sparsity |  |
|  | R: Changes to Collaboration: interdisciplinary, cross-sectoral |  |
|  | R: Changes to Resources: financial, tangible, labour |  |
|  | R: Changes to Access |  |
|  | R: Changes to Equity |  |
| **Year** | 2018, 2019, 2020, 2021, 2022, 2023 | Year of publication |
| **Population: Socio-demographic Group** | NA | Adapted from COCHRANE+ groups used in equity section. Papers that did not specify a specific population were considered “General”. Papers that examined non-human aspects of FSNH (i.e. livestock or plant health) were coded as “NA”. |
|  | General |  |
|  | Age (i.e. children, youth, older) |  |
|  | Gender |  |
|  | Race, Ethnicity, Culture, Language |  |
|  | Religion |  |
|  | Education |  |
|  | Socioeconomic Status |  |
|  | Occupation (O) |  |
|  | O: Farmers, Land owners |  |
|  | O: Pastoralists, Herders |  |
|  | Place of Residence (PR) |  |
|  | PR: Rural, Urban |  |
|  | PR: Migrants, Nomads |  |
|  | PR: Refugees, Conflict affected populations |  |
| **Setting: Region** | Global | Based on World Bank regions. Papers that did not specify a geographical setting were considered “Global”. |
|  | East Asia and Pacific |  |
|  | Europe and Central Asia |  |
|  | Latin America and Caribbean (LAC) |  |
|  | Middle East and North Africa (MENA) |  |
|  | North America |  |
|  | South Asia |  |
|  | Sub-Saharan Africa (SSA) |  |
| **Setting: Economies** | NA | Based on World Bank regions country income classifications and internationally recognised economic groups. Papers that did not specify an economic setting were coded as “NA”. |
|  | LMIC: Low and Middle-Income Countries |  |
|  | UMIC: Upper Middle Income Countries |  |
|  | HIC: High Income Countries |  |
|  | OECD: Organisation for Economic Co-operation and Development countries |  |
|  | BRICS: Brazil, Russia, India, China, and South Africa |  |
|  | SIDS: Small Island Developing States |  |
|  | EU: European Union |  |
| **Setting: Agroecological Zones** | NA | Adapted from FAO agroecological zone classifications. Papers that did not specify an agroecological zone were coded as “NA”. |
|  | Arid & Semi-arid (i.e. hot/cold, dry) |  |
|  | Tropical (i.e. hot, humid) |  |
|  | Sub-tropical (i.e. Mediterranean) |  |
|  | Temperate & Alpine |  |
|  | Arctic & Boreal |  |
| **Setting: Country** | … | We used World Bank names for country specific papers. Papers that did not specify country were coded as NA. |

#### C. Supplemental Methods 4C: Sub-domains that were automatically cross-coded to address thematic overlaps

| **THEMATIC AREA** | **SUB-THEMES** | **AUTOMATIC ADDITIONAL CODES*** |
| --- | --- | --- |
| **Type of Review (Study Design)** | |  |
| **Directionality (domain related)** | |  |
| **Domain: Climate Change** | |  |
| **Domain: Food Systems, Nutrition & Health (FSNH)** | *FSNH Domain: Primary Food Production* | |
|  | CP: Herbs, Teas, Coffee, Cacao | FSNH-ENV: Biodiversity, Agroecology, Agroforestry |
|  | ASF: Cattle | FSNH-ASF: Ruminant Livestock |
|  | ASF: Goats, Sheep | FSNH-ASF: Ruminant Livestock |
|  | ASF: Dairy | FSNH-ASF: Ruminant Livestock;  FSNH-ASF: Cattle AND/OR Goats, Sheep (as applicable) |
|  | *FSNH Domain: Food Security & Diets* | |
|  | Infant & Child Feeding (IYCF) | Diets |
|  | *FSNH Domain: Nutrition-related Health* | |
|  | MAL: Infant Growth (i.e. PTB, SGA, wasting, stunting) | FSNH-MAL: Undernutrition |
| **Strategy (mechanism related)** | |  |
| **Mechanism of Change (MoC)** | *MoC: Knowledge Attitudes Practices (KAP)* | |
|  | P: GHG Fixation, Sequestration | Specific CC-GHG code (as applicable) |
| **Equity** | |  |
| **Key Recommendations** | |  |
| **Year** | |  |
| **Population: Socio-demographic Group** | |  |
| **Setting: Region** | |  |
| **Setting: Economies** | |  |
| **Setting: Agroecological Zones** | |  |
| **Setting: Country** | | Setting: Region & Economies (using World Bank definitions) |
| * Please note that while many other codes overlap, only these few were consistently considered inherent members of other group. This flexible approach allowed for more accurate coding of more niche papers. | | |

### Supplemental Results 1: Full list of all key literature suggested by experts (not all included as only 14 met inclusion criteria)

| **#** | **Type** | **Reference** | **Include/ Exclude** | **Within Search** | **Notes** | **Experts** |
| --- | --- | --- | --- | --- | --- | --- |
| 1 | Review | Duchenne-Moutien Ramona A, Neetoo Hudaa. Climate Change and Emerging Food Safety Issues: A Review. Journal of food protection. 2021;84(11):1884–97. | Include | Yes |  | 13 |
| 2 | Review | El Khayat M, Halwani DA, Hneiny L, Alameddine I, Haidar MA, Habib RR. Impacts of Climate Change and Heat Stress on Farmworkers’ Health: A Scoping Review. Frontiers in Public Health [Internet]. 2022;10. Available from: https://www.frontiersin.org/articles/10.3389/fpubh.2022.782811 | Include | Yes |  | 15 |
| 3 | Review | Farooq MS, Uzair M, Raza A, Habib M, Xu Y, Yousuf M, et al. Uncovering the Research Gaps to Alleviate the Negative Impacts of Climate Change on Food Security: A Review. Frontiers in Plant Science [Internet]. 2022;13. Available from: https://www.frontiersin.org/articles/10.3389/fpls.2022.927535 | Include | Yes |  | 14 |
| 4 | Review | Scheelbeek PFD, Bird FA, Tuomisto HL, Green R, Harris FB, Joy EJM, et al. Effect of environmental changes on vegetable and legume yields and nutritional quality. Proceedings of the National Academy of Sciences. 2018 Jun;115(26):6804–9. | Include | Yes |  | 1 |
| 5 | Review | Birgani RA, Kianirad A, Shab-Bidar S, Djazayeri A, Pouraram H, Takian A. Climate Change and Food Price: A Systematic Review and Meta-Analysis of Observational Studies, 1990-2021. American Journal of Climate Change. 2022;11(02):103–32. | Include | No | Not indexed in databases | 15 |
| 6 | Review | Fanzo J, Davis C, McLaren R, Choufani J. The effect of climate change across food systems: Implications for nutrition outcomes. Global Food Security. 2018;18:12–9. | Include | No | "Review" terms not in abstract | 4 |
| 7 | Review | Fanzo J, Haddad L, Schneider KR, Béné C, Covic NM, Guarin A, et al. Viewpoint: Rigorous monitoring is necessary to guide food system transformation in the countdown to the 2030 global goals. Food Policy. 2021;104:102163–102163. | Include | No | "Review" terms not in abstract | 14 |
| 8 | Review | Mora C, McKenzie T, Gaw IM, Dean JM, von Hammerstein H, Knudson TA, et al. Over half of known human pathogenic diseases can be aggravated by climate change. Nature Climate Change. 2022;12(9):869–75. | Include | No | "FSNH" or "review" terms not in abstract | 4 |
| 9 | Review | Myers S, Fanzo J, Wiebe K, Huybers P, Smith M. Current guidance underestimates risk of global environmental change to food security. BMJ. 2022 Sep 29;378:e071533. | Include | No | "Review" terms not in abstract | 17 |
| 10 | Review | Swinburn BA, Kraak VI, Allender S, Atkins VJ, Baker PI, Bogard JR, et al. The Global Syndemic of Obesity, Undernutrition, and Climate Change: The Lancet Commission report. The Lancet. 2019 Feb;393(10173):791–846. | Include | No | Doesn't have an abstract | 4, 13 |
| 11 | Review | Willett W, Rockström J, Loken B, Springmann M, Lang T, Vermeulen S, et al. Food in the Anthropocene: the EAT–Lancet Commission on healthy diets from sustainable food systems. The Lancet. 2019 Feb;393(10170):447–92. | Include | No | Doesn't have an abstract | 8 |
| 12 | Grey literature | Bush A, Wrottesley S, Mates E, Fenn B. Nutrition and Climate Change - Current State of Play: Scoping Review. 2022. | Include | No | Not indexed in databases | 13, 14 |
| 13 | Grey literature | Ciucci M, Cujkova J. Climate change and its impact on food and nutrition security. Euopean Parliament - ENVI committee; 2020. | Include | No | Didn't search the EU Commission database | 14 |
| 14 | Grey literature | Lewis Y, Gower A, Notten P. Single-use beverage cups and their alternatives: Recommendations from Life Cycle Assessments - Life Cycle Initiative [Internet]. United Nations Environment Programme; 2021 Feb [cited 2023 Oct 2]. Available from: https://www.lifecycleinitiative.org/library/single-use-beverage-cups-and-their-alternatives-lca/, https://www.lifecycleinitiative.org/library/single-use-beverage-cups-and-their-alternatives-lca/ | Include | No | Didn't search UN Databases | 12 |
| 15 | Grey literature | Lewis Y, Gower A, Notten P. Single-use plastic tableware and its alternatives: Recommendations from Life Cycle Assessments - Life Cycle Initiative [Internet]. United Nations Environment Programme; 2021 Feb [cited 2023 Oct 2]. Available from: https://www.lifecycleinitiative.org/library/single-use-plastic-tableware-and-its-alternatives-recommendations-from-life-cycle-assessments-2/, https://www.lifecycleinitiative.org/library/single-use-plastic-tableware-and-its-alternatives-recommendations-from-life-cycle-assessments-2/ | Include | No | Didn't search UN Databases | 12 |
| 16 | Grey literature | Miliutenko S, Sandin G, Liptow C. Single-use plastic take-away food packaging and its alternatives - Life Cycle Initiative [Internet]. United Nations Environment Programme; 2020 Oct [cited 2023 Oct 2]. Available from: https://www.lifecycleinitiative.org/library/single-use-plastic-take-away-food-packaging-and-its-alternatives/, https://www.lifecycleinitiative.org/library/single-use-plastic-take-away-food-packaging-and-its-alternatives/ | Include | No | Didn't search UN Databases | 12 |
| 17 | Grey literature | Notten P, Lewis Y, Burke M, Corella-Puertas E, Boulay AM. Single-use supermarket food packaging and its alternatives: Recommendations from Life Cycle Assessments - Life Cycle Initiative [Internet]. United Nations Environment Programme; 2022 [cited 2023 Oct 2]. Available from: https://www.lifecycleinitiative.org/library/single-use-supermarket-food-packaging-and-its-alternatives-recommendations-from-life-cycle-assessments/, https://www.lifecycleinitiative.org/library/single-use-supermarket-food-packaging-and-its-alternatives-recommendations-from-life-cycle-assessments/ | Include | No | Didn't search UN Databases | 12 |
| 18 | Grey literature | United Nations Environment Programme. Single-use plastic bottles and their alternatives: Recommendations from Life Cycle Assessments - Life Cycle Initiative [Internet]. 2020 Jul [cited 2023 Oct 2]. Available from: https://www.lifecycleinitiative.org/library/single-use-plastic-bottles-and-their-alternatives-recommendations-from-life-cycle-assessments/, https://www.lifecycleinitiative.org/library/single-use-plastic-bottles-and-their-alternatives-recommendations-from-life-cycle-assessments/ | Include | No | Didn't search UN Databases | 12 |
| 19 | Review | Altieri MA, Nicholls CI. The adaptation and mitigation potential of traditional agriculture in a changing climate. Climatic Change. 2017;140(1):33–45. | Exclude | No | Cannot Access | 13 |
| 20 | Review | Bryan E, Theis S, Choufani J, De Pinto A, Suseela Meinzen-Dick R, Ringler C. Conceptual framework: Gender, climate change, and nutrition integration initiative. 2017. | Exclude | No | Not within timeframe | 14 |
| 21 | Review | Eriksen S, Aldunce P, Bahinipati CS, Martins RD, Molefe JI, Nhemachena C, et al. When not every response to climate change is a good one: Identifying principles for sustainable adaptation. Climate and Development. 2011 Jan;3(1):7–20. | Exclude | No | Not within timeframe | 18 |
| 22 | Review | Fruttero A, Halim D, Broccolini C, Coelho B, Gninafon H, Muller N. Gendered Impacts of Climate Change. World Bank. 2023; | Exclude | No | No link to FSNH | 15 |
| 23 | Review | Gustafson D, Gutman A, Leet W, Drewnowski A, Fanzo J, Ingram J. Seven Food System Metrics of Sustainable Nutrition Security. SUSTAINABILITY. 2016;8(3). | Exclude | No | Not within timeframe | 14 |
| 24 | Review | Masters WA. Review of Global Warming and Agriculture: Impact Estimates by Country. Journal of Economic Literature. 2008;46(2):448–50. | Exclude | No | Not within timeframe | 3 |
| 25 | Review | Müller C, Elliott J, Levermann A. Fertilizing hidden hunger. Nature Climate Change. 2014;4(7):540–1. | Exclude | No | Not within timeframe | 13 |
| 26 | Review | Myers SS, Smith MR, Guth S, Golden CD, Vaitla B, Mueller ND, et al. Climate Change and Global Food Systems: Potential Impacts on Food Security and Undernutrition. Annual Review of Public Health. 2017 Mar;38(1):259–77. | Exclude | No | Not within timeframe | 4, 13 |
| 27 | Review | Nelson G, Bogard J, Lividini K, Arsenault J, Riley M, Sulser TB, et al. Income growth and climate change effects on global nutrition security to mid-century. Nature Sustainability. 2018;1(12):773–81. | Exclude | No | Not a review (modelling) | 8 |
| 28 | Review | O’Brien K, Eriksen S, Nygaard LP, Schjolden A. Why different interpretations of vulnerability matter in climate change discourses. Climate Policy [Internet]. 2007 Jan 1 [cited 2023 Oct 2]; Available from: https://www.tandfonline.com/doi/abs/10.1080/14693062.2007.9685639 | Exclude | No | Not within timeframe | 18 |
| 29 | Review | Taub DR, Miller B, Allen H. Effects of elevated CO2 on the protein concentration of food crops: a meta-analysis. Global Change Biology. 2008 Mar;14(3):565–75. | Exclude | No | Not within timeframe | 13 |
| 30 | Review | Tubiello F, Rosenzweig C. Developing climate change impact metrics for agriculture. Integrated Assessment Journal. 2008 Jun;8(1). | Exclude | No | Not within timeframe | 14 |
| 31 | Review | Walton S, Hawkes C, Fanzo J. Searching for the essential: Exploring practitioners’ views on actions for re-orienting food systems towards healthy diets. Global Food Security. 2023;37:100687–100687. | Exclude | No | Not related to CC | 13 |
| 32 | Review | An R, Ji M, Zhang S. Global warming and obesity: a systematic review. Obesity Reviews. 2018;19(2):150–63. | Exclude | Yes | Not within time frame (pub date 2017) | 13 |
| 33 | Review | Poore J, Nemecek T. Reducing food’s environmental impacts through producers and consumers. Science. 2018 Jun;360(6392):987–92. | Exclude | No | Erratum | 1 |
| 34 | Primary research | Battilani P, Toscano P, Van der Fels-Klerx HJ, Moretti A, Leggieri MC, Brera C, et al. Aflatoxin B-1 contamination in maize in Europe increases due to climate change. SCIENTIFIC REPORTS. 2016;6. | Exclude | No | Not a review (modelling) | 13 |
| 35 | Primary research | Beach RH, Sulser TB, Crimmins A, Cenacchi N, Cole J, Fukagawa NK, et al. Combining the effects of increased atmospheric carbon dioxide on protein, iron, and zinc availability and projected climate change on global diets: a modelling study. The Lancet Planetary Health. 2019;3(7):e307–17. | Exclude | No | Not a review (modelling) | 8 |
| 36 | Primary research | Geyik Ö, Hadjikakou M, Bryan BA. Climate-friendly and nutrition-sensitive interventions can close the global dietary nutrient gap while reducing GHG emissions. Nature Food. 2023;4(1):61–73. | Exclude | No | Not a review (modelling) | 14 |
| 37 | Primary research | Mehrabi Z, Delzeit R, Ignaciuk A, Levers C, Braich G, Bajaj K, et al. Research priorities for global food security under extreme events. One Earth. 2022 Jul;5(7):756–66. | Exclude | No | Not a review (survey) | 4 |
| 38 | Primary research | Mulmi P, Block SA, Shively GE, Masters WA. Climatic conditions and child height: Sex-specific vulnerability and the protective effects of sanitation and food markets in Nepal. Economics & Human Biology. 2016;23:63–75. | Exclude | No | Not a review | 3 |
| 39 | Primary Research | Rosenzweig C, Tubiello FN. Developing Climate Change Impacts and Adaptation Metrics for Agriculture. 2006; | Exclude | No | Not within timeframe | 14 |
| 40 | Primary research | Springmann M, Clark M, Mason-D’Croz D, Wiebe K, Bodirsky BL, Lassaletta L, et al. Options for keeping the food system within environmental limits. Nature. 2018;562(7728):519–25. | Exclude | No | Not a review (modelling) | 8 |
| 41 | Primary research | Sulser TB, Beach RH, Wiebe KD, Dunston S, Fukagawa NK. Disability-adjusted life years due to chronic and hidden hunger under food system evolution with climate change and adaptation to 2050. The American Journal of Clinical Nutrition. 2021 Aug;114(2):550–63. | Exclude | No | Not a review (modelling) | 8 |
| 42 | Primary research | Xu X, Sharma P, Shu S, Lin TS, Ciais P, Tubiello FN, et al. Global greenhouse gas emissions from animal-based foods are twice those of plant-based foods. Nature Food. 2021;2(9):724–32. | Exclude | No | Not a review (modelling) | 13 |
| 43 | Grey literature | Bezner Kerr R, Hasegawa T, Lasco R, Bhatt I, Deryng D, Farrell A, et al. Food, Fibre, and Other Ecosystem Products. In: Intergovernmental Panel On Climate Change (Ipcc), editor. Climate Change 2022 – Impacts, Adaptation and Vulnerability: Working Group II Contribution to the Sixth Assessment Report of the Intergovernmental Panel on Climate Change. Cambridge: Cambridge University Press; 2022. | Exclude | No | Book chapter (but very big one) - beyond scope | 4, 11 |
| 44 | Grey literature | Mbow C, Rosenzweig C, Barioni TG, Herrero M, Krishnapillai M, Liwenga E, et al. Food Security. In: Intergovernmental Panel on Climate Change (IPCC), editor. Climate Change and Land: IPCC Special Report on Climate Change, Desertification, Land Degradation, Sustainable Land Management, Food Security, and Greenhouse Gas Fluxes in Terrestrial Ecosystems [Internet]. Cambridge: Cambridge University Press; 2019. p. 437–550. Available from: https://www.cambridge.org/core/books/climate-change-and-land/food-security/AB0D996CA6E915F6EAF7A2EEB77AD09D | Exclude | No | Book chapter (but very big one) - beyond scope | 4, 11 |
| 45 | Grey literature | Bélanger J, Pilling D. The state of the world’s biodiversity for food and agriculture [Internet]. Rome, Italy: FAO Commission on Genetic Resources for Food and Agriculture; 2019. Available from: http://www.fao.org/3/CA3129EN/CA3129EN.pdf | Exclude | No | Not a review (book report) | 13 |
| 46 | Grey literature | FAO, WHO. Sustainable healthy diets – Guiding principles [Internet]. Rome, Italy: FAO and WHO; 2019 [cited 2023 Oct 2]. Available from: https://www.fao.org/policy-support/tools-and-publications/resources-details/en/c/1329630/ | Exclude | No | Analytical link between CC and FSNH | 13 |
| 47 | Grey literature | Global Nutrition Report. Global Nutrition Report: The state of global nutrition [Internet]. Global Nutrition Report; 2022. Available from: https://globalnutritionreport.org/reports/2022-global-nutrition-report/ | Exclude | No | Not a review (book report) | 13 |
| 48 | Grey literature | Loken B, McFeely P. Solving the Great Food Puzzle: 20 levers to scale national action. Gland, Switzerland: WWF; 2022. | Exclude | No | Not a review (book report) | 13 |
| 49 | Grey literature | McLaren S, Berardy A, Henderson A, Holden N, Huppertz T, Jolliet O, et al. Integration of environment and nutrition in life cycle assessment of food items: opportunities and challenges. 2021. | Exclude | No | Not a review (book report) | 12 |
| 50 | Grey literature | United Nations Environment Programme. Single-use plastic bags and their alternatives: Recommendations from Life Cycle Assessments - Life Cycle Initiative [Internet]. 2020 Aug [cited 2023 Oct 2]. Available from: https://www.lifecycleinitiative.org/library/single-use-plastic-bags-and-their-alternatives-recommendations-from-life-cycle-assessments/, https://www.lifecycleinitiative.org/library/single-use-plastic-bags-and-their-alternatives-recommendations-from-life-cycle-assessments/ | Exclude | No | Analytical link between CC and FSNH | 12 |
| 51 | Grey literature | World Bank Group. World development report 2010 : development and climate change. Bierbaum R, Fay M, Ross-Larson B, editors. Washington D.C.: World Bank Group; 2010. | Exclude | No | Not within timeframe | 1 |
| 52 | Grey literature (report) | Chen DX, McDonald DK, Rose M, Wright J, Portley N, Michalakis M. Stemming the Plastic Climate Crisis [Internet]. Pacific Environment; 2023. Available from: https://www.pacificenvironment.org/wp-content/uploads/2023/05/Stemming-the-Plastic-Climate-Crisis-1.pdf | Exclude | No | Analytical link between CC and FSNH | 12 |
| 53 | Database | SimaPro. SimaPro. 2023 [cited 2023 Oct 2]. AGRIBALYSE 3.1 French agricultural and food database. Available from: https://simapro.com/products/agribalyse-agricultural-database/ | Exclude | No | Not peer-reviewed or published literature. | 12 |
| 54 | Database | SimaPro. SimaPro. 2023 [cited 2023 Oct 2]. Agri-footprint database (additional downloads) (additional download). Available from: https://simapro.com/products/agri-footprint-mass-energy/ | Exclude | No | Not peer-reviewed or published literature. | 12 |
| 55 | Database | SimaPro. SimaPro. 2023 [cited 2023 Oct 2]. Environmental Footprint database. Available from: https://simapro.com/products/environmental-footprint-database/ | Exclude | No | Not peer-reviewed or published literature. | 12 |
| 56 | Database | SimaPro. SimaPro. 2023 [cited 2023 Oct 2]. ESU world food LCA database - SimaPro database. Available from: https://simapro.com/products/esu-world-lca-food-database/ | Exclude | No | Not peer-reviewed or published literature. | 12 |
| 57 | Database | SimaPro. SimaPro. 2023 [cited 2023 Oct 2]. Quantis World Food LCA Database. Available from: https://simapro.com/products/quantis-world-food-lca-database/ | Exclude | No | Not peer-reviewed or published literature. | 12 |
| 58 | Database | USDA. Life Cycle Assessment: Resources [Internet]. 2023. Available from: https://data.nal.usda.gov/life-cycle-assessment | Exclude | No | Not peer-reviewed or published literature. | 12 |
| 59 | Webpage | European Comission. Biodiversity strategy for 2030 [Internet]. 2023 [cited 2023 Oct 2]. Available from: https://environment.ec.europa.eu/strategy/biodiversity-strategy-2030_en | Exclude | No | Not peer-reviewed or published literature. | 13 |
| 60 | Webpage | European Comission. EU-Africa: Global Gateway Investment Package [Internet]. 2021 [cited 2023 Oct 2]. Available from: https://commission.europa.eu/strategy-and-policy/priorities-2019-2024/stronger-europe-world/global-gateway/eu-africa-global-gateway-investment-package_en | Exclude | No | Not peer-reviewed or published literature. | 13 |
| 61 | Webpage | FAO. Alternative proteins top the bill for the latest FAO–International Sustainable Bioeconomy Working Group webinar \| Sustainable and circular bioeconomy for food systems transformation \| Food and Agriculture Organization of the United Nations [Internet]. 2022 [cited 2023 Oct 2]. Available from: https://www.fao.org/in-action/sustainable-and-circular-bioeconomy/resources/news/details/en/c/1507553/ | Exclude | No | Not peer-reviewed or published literature. | 13 |
| 62 | Webpage | Management of Conflicts of Interests in Public-Private Partnerships [Internet]. 2022 [cited 2023 Oct 2]. Available from: https://www.youtube.com/watch?v=spC-JDnxNtY | Exclude | No | Not peer-reviewed or published literature. | 13 |
| 63 | Webpage | Rees E. CropLife International. 2023 [cited 2023 Oct 2]. Advancing Innovation in Agriculture for Climate Impact. Available from: https://croplife.org/blog-innovation-for-climate-impact/ | Exclude | No | Not peer-reviewed or published literature. | 13 |
| 64 | Webpage | World Bank Group. World Bank. 2021 [cited 2023 Oct 2]. Climate-Smart Agriculture. Available from: https://www.worldbank.org/en/topic/climate-smart-agriculture | Exclude | No | Not peer-reviewed or published literature. | 13, 14 |
| 65 | Webpage | World Economic Forum. World Economic Forum. 2021 [cited 2023 Oct 2]. What is ‘nature positive’ and why is it the key to our future? Available from: https://www.weforum.org/agenda/2021/06/what-is-nature-positive-and-why-is-it-the-key-to-our-future/ | Exclude | No | Not peer-reviewed or published literature. | 13 |
| 66 | Other | Life Cycle Initiative. Life Cycle Initiative. 2023 [cited 2023 Oct 2]. Reports & Training Materials - Life Cycle Initiative. Available from: https://www.lifecycleinitiative.org/library/, https://www.lifecycleinitiative.org/library/ | Exclude | No | Not peer-reviewed or published literature. | 12 |
| 67 | Other | NIH. NIH. 2023 [cited 2023 Oct 2]. ADVANTAGE (Agriculture and Diet: Value Added for Nutrition, Translation, and Adaptation in a Global Ecology) Project Virtual Meeting Series \| NICHD - Eunice Kennedy Shriver National Institute of Child Health and Human Development. Available from: https://www.nichd.nih.gov/about/meetings/2023/041423 | Exclude | No | Not peer-reviewed or published literature. | 12 |

### Supplemental Results 2: Full analysis of the expert consultation

This supplement aims to provide readers with the richer and more extensive details of consultation analysis. The key take-aways of this consultation are summarized in the main text.

*Evidence Gaps:*

Specific research gaps in FSNH domains linked to climate change shown in the consultation contextualised the results of the EGM further. This included under-researched sub-sectors including the food system’s post-harvest, distribution, retail, consumption stages. Several respondents mentioned a lack of understanding the implications of dramatic shifts in diets due to globalised systems in concert with climate change, as the agricultural systems in low-income settings evolved particularly to support dietary patterns of those in high-income settings. Many mentioned various aspects of stressors and shocks vis a vis food systems and health. A few respondents mentioned the focus on markets and retailers, questioning their role in suppling healthy diets amidst a climate-affected landscape. One respondent said, “The majority of food retailers believe they can adapt their supply chains very quickly but we actually do not know the stress limits of this or how it might affect nutrition and health in future” (Respondent 1). Another commented, “There has been loads of work focusing on the production side in terms of sustainability and adaptation but very little on the consumer side” (Respondent 18). Mental health related to food and climate was highlighted as a critical research gap related to stressors and shocks. Another respondent pointed to the conflicting role of processed foods, on one hand reducing food loss and waste, on another contributing to exponentially rising diet-related non-communicable diseases. Additionally, understanding the connections between climate change, agriculture, and infectious disease risks in LMICs and incorporating Water, Sanitation, and Hygiene (WASH) considerations into agricultural strategies to reduce infectious disease risks were also raised by experts.

Many experts noted the lack of understanding of dynamic, interacting, and complex risks and impacts that characterise the relationship between climate change and FSNH. This included research accounting for interactions with broad socio-political and economic factors, behavioural elements of food choice, and other environmental conditions. “Most of the literature focuses on fairly simple pathways (e.g. heat stress affecting food production) rather than more complex interactions such as climate change compounding other drivers which affect agriculture, nutrition and health, or having unanticipated outcomes” (Respondent 9).

Respondents contrasted one another about trade-offs between breadth and depth in research approaches. Some argued that Big Data and global analyses would have the most impact on planning mitigation and adaptation strategies. Others argued that much more granular or localised information would be necessary to allow for effective and time-sensitive action. For instance, proponents of a more nuanced approach suggest analyses would be more useful if they were at national and especially subnational scales, on different population characteristics, and included more localised contextual factors. One respondent pointed to problems with outdated data, such as food consumption tables. Evidence suggesting a stark decline in the nutrient density of foods due to climate change means that using old reference points will not inform effective solutions. A few respondents said that the uncertainty of existing models was a significant gap. For instance, even well-researched models quantifying the climate change impacts on crop yields and productivity point to some positives and some negatives, but not enough confidence in their precision or predictive capacity. Many respondents noted the lack of tools, metrics and methods needed to achieve integration in data, analysis, collaboration and action.

Respondents mentioned a lack of decision-making tools to inform near-term research and policy implementation. They often cited inaccessible data, such as a lack of data or of open-source data, and a dearth of capacity to combine, analyse and utilise complex data for decision-making. Several respondents noted the complexities (and sometimes the potential) of engaging with the private sector, especially to gain access to additional and better data. “We need to identify how to incentivize the private sector, who will lose out on CSA and agricultural transformation, and how this will impact vulnerable populations worldwide.” (Respondent 5). Several respondents alluded to or argued explicitly that many barriers at this nexus are political, economic, and strategic. Several pointed out missing approaches in identifying catalysts and levers for meaningful change, especially in the socio-political space (e.g. via political economy analysis).

Vulnerability was mentioned frequently, albeit in different contexts. Experts noted misconceptions or lack of clarity about drivers of, and more importantly, approaches to respond to, vulnerability overall. Some points about vulnerability were not directly related to equity concerns, whereas others were. Regarding the former, they mentioned the lack of knowledge on vulnerabilities of the food supply chain (especially due to globalization). Some experts mentioned complex risks, specifically how risks can cascade across sectors and systems, like the water-food-energy-health-biodiversity nexus.

In terms of equity, the vulnerability of specific populations was mentioned in several ways, including the differential impacts of climate change on FSNH for populations already marginalized (e.g. by remoteness and sociodemographic differences). Several respondents mentioned migration. One respondent highlighted worsening FSNH outcomes for people who are unable to migrate from regions acutely impacted by climate change: “The basic story of migration from the evidence so far is that the poorest and most vulnerable move very little – for them there will be more climate-poverty traps than there will be climate refugees” (Respondent 3). Another said that the migration of adolescent boys and young men was not getting enough attention because they aren’t seen as vulnerable, but “from a family systems perspective, mainly young male migration disrupts family systems. Migration itself is a massive change in food environments and diets” (Respondent 16). Some respondents referred generally to climate contributing to downward spirals of vulnerability and entrapment, and the care that would be needed to ensure response would be equitable.

A few respondents further linked vulnerability and equity to the overarching narratives of these problems and corresponding gaps in methodological approaches. The “vulnerability of any system has different standpoints, such as 'outcome vulnerability' and 'contextual vulnerability'. Scientific discourse often deals only with 'outcome vulnerability' and drives policies and programs according to that framing. On the other hand, 'contextual vulnerability' deals with local and intersectional factors which play significant roles in determining the vulnerability of a system.  Vulnerabilities to climate change [go] beyond the physical damages and vary per household’s structural and social intersections such as ethnic and religious status, caste, economic conditions, or geographic locations. However, this discourse is less represented in the adaptation literature and in the adaptation process itself” (Respondent 17). Another said, “True validation of…indicators commonly used to proxy for dimensions of climate, planet, food system, health, economics, justice dimensions is still needed” (Respondent 10), and several mentioned wanting to re-think social justice dimensions of metrics and methods, how these are applied, and what guidance might be needed to ensure equitable approaches and actions.

*Areas for future investment:*

Several respondents mentioned the need to identify which kinds of data, research methods and approaches would inform food system action. Some questioned whether macro- or micro-systems focus would be most informative for identifying levers of change. One respondent noted that most models are regional or national but don’t give insight into localised vulnerabilities, whether geographic, socio-economic, cultural, or political. One respondent argued that differing understandings of concepts such as ‘sustainability’ will change their operational response: “There seem to be two narratives on climate change, either causes (High-Income Countries) or consequences (LMIC)” (Respondent 16). Some noted that methods are more evolved to measure the impacts of acute events or disasters but much less evolved for slower (but no less profound) changes such as pest infestation, non-acute flooding, or temperature changes. The inability to capture the spatial-temporal aspects of changing climates, food systems and health poses challenges to planning effective interventions.

Some suggested more political economy and econometric analysis to identify the most effective actions in sustainably transforming the food system. Respondent 5 said “A missing piece is Economics [and] cost modelling, capturing both nutrition sensitive and climate sensitive aspects…A huge question politically is what’s it going to cost, how long will it take, what will transform most. For instance, in the IPCC synthesis report there are lots of solutions, [stating] who is going to do what and when. In food systems, the soft political side is missing…From decades of action on multisectoral nutrition, [we see that] technocrats ended up controlling the narrative, and we are in danger of going down that road again.”

Other respondents focused on the nature of the research community, arguing that integrating communities of practice on climate and FSNH issues would result in clearer, actionable evidence. Some suggested developing tools, metrics, and methods able to achieve integration. They noted limited research that truly combines disciplines and methods, for example, combinations of qualitative, modelling, and spatial tools. One respondent mentioned the need for user-friendly, publicly available tools that combine GIS and other data types. A few respondents mentioned methodologies such as system dynamics, noting the dearth of application on climate-FSNH themes and a general lack of sophistication in food systems and health research compared to climate research.

Respondents highlighted the promise of participatory and scenario tools combined with epidemiological analysis, system dynamics modelling and Life Cycle Analysis (LCA). One respondent acknowledged LCA was well-represented in the EGM but suggested that emerging innovations on LCA would make them more meaningful, e.g., consequential LCA, or accounting for dynamic future changes and trade-offs, spatially disaggregated impact assessment, and combining LCA with planetary boundary framework approaches. Several respondents mentioned the plethora of tools in the climate-FSNH space, but common limiting factors such as the need for large data inputs or high complexity. Contrastingly, both data complexity/data intensity (too much data) and data deficits were mentioned as barriers to research and decision-making efforts. Experts emphasized the need for tools that are accessible and user-friendly to allow for equitable participation and informed decision-making across different sectors and groups. This supports an overall conclusion from the consultation that there are two different gaps to fill: a) models and analyses built by experts that include much more data and types of data and, critically, have better precision and predictive capacity even in the face of changing scenarios; and b) effective tools that are straightforward, easy and free to use by non-experts, that have enough granularity for decision-making at a sub-national level or above.

Some experts mentioned approaches or principles that would make any actions both more equitable and effective, while concurrently making progress on adaptation and mitigation. One expert suggested integrating feminist principles into any effort to foster leadership and actions that promote health of both people and nature. Another respondent highlighted the need for bottom-up, participatory approaches and co-produced solutions. Experts also identified certain principles as key to connecting domains of FSNH. For example, two consultants highlighted principles of biodiversity and agroecology in improving health outcomes through enhanced crop productivity and nutritional quality.

### Supplemental Results 3: Thematic research gaps identified through expert consultation, grouped by relevance to adaptation, mitigation, or both (adaptation and mitigation).

| **NOTE: These consultation responses are presented as direct quotation or faithful summary of statements made by respondents (numbered to maintain anonymity).**  *Adaptation*  Food Systems: “One major gap is the understanding of food value chains and trade patterns and how these respond to climate shocks. We know very little about the vulnerability of food systems to climate change because trade is so globalized and complicated. The majority of food retailers believe they can adapt their supply chains very quickly but we actually do not know the stress limits of this or how it might affect nutrition and health in future. To understand it better we would need detailed supply chain data and probably complex econometric models, but the data will be quite hard to come by without industry involvement.” (Respondent 1, echoed by Respondent 5)  Migration: “Migration related to climate is a growing problem at both sub-national (especially rural-to-urban) and international (especially from more tropical to more temperate) levels.  People often say there will be “climate refugees” but that is not what actually happens.  Most actual refugees are displaced by conflict, because only violence is a severe enough threat to give people no choice but to give up everything and go.  However, such conflicts are often a result of resource scarcity, which is directly linked to climate change. The basic story of migration from the evidence so far is that the poorest and most vulnerable move very little – for them there will be more climate-poverty traps than there will be climate refugees. There is a gap in research in understanding these migration patterns and addressing these vulnerable populations (18).” (Respondent 3)  Nutritional value of crops: “Despite a large volume of literature in the area, the impacts of climate change on crop productivity and especially crop nutritional content are still highly uncertain. Crop yield estimates under high-emission scenarios are highly uncertain due to the CO_2_ effect in the models, and other constraining factors such as hastened phenological development, which is represented very differently across models. The representation of nutritional quality of crops is even more uncertain, not just in the models, but also among experimental data. Higher CO_2_ levels “dilute" crop yields and lead to lower nutritional density, but higher temperatures offset some of these effects and the balance of the two driving factors under future climate change scenarios remains highly uncertain with very limited evidence across agroecosystems.” (Respondent 7)  Extreme Events: “The long-term changes and impacts of climate variability and extreme weather on FSNH is unclear. While there is work being done in this area, not a lot of it is linking it to nutrition and health.” (Respondent 8)  Health: Links between climate change, agriculture, and infectious disease risks are currently unclear, particularly in LMICs. Future research is needed on how WASH can be integrated into agricultural strategies, particularly animal agriculture in low-resource settings, to reduce infectious disease risk and morbidity. (Respondent 4)  *Mitigation*  Food transport: Middle parts of the chain are a huge research gap, such as commodity structures, processing and transport as most of us eat food-like substances. These parts of the food chain can be modelled in a much more rigorous way, particularly transportation as a contributor to or mitigator of climate change. (Respondent 5)  Food systems and diets: Agriculture and food systems have an outsized impact on climate change and the adoption of environmentally sustainable diets globally is urgently needed to mitigate the negative health effects of climate change. Research is needed to understand what a healthy, environmentally sustainable diet looks like across different cultures and populations groups (Respondent 4)  *Both*  Climate-Smart Agriculture (CSA): Climate-resilient agricultural strategies can both enable households/individuals to adapt to climate change and can contribute to climate change mitigation. “Research gaps include understanding which strategies are adaptable to various contexts (particularly those most vulnerable to climate change), their impacts on nutrition and health outcomes, and equity outcomes.” (Respondent 4). “We need identify how to incentivize private sector, who will lose out on CSA and agriculture transformation, and this will impact vulnerable populations world-wide.” (Respondent 5)  Indigenous & Local Knowledge (ILK): “There is a gap in integrating ILK into research on climate change adaptation and mitigation (climate-resilient agriculture) and policies. Indigenous communities are particularly vulnerable to the effects of climate change, and also hold important knowledge on climate resilience. Understanding how to integrate ILK into research including methods and best practices is important for equity and research progress”. (Respondent 4)  Biodiversity: Biodiversity plays a critical role in nutrition and health, and mitigation strategies account for this. Agriculture is highly dependent on biodiversity, and the urgent biodiversity crisis warrants understanding how losses in biodiversity will affect nutrition and health outcomes. Research is also needed on how agriculture and food can be a lever to mitigate biodiversity loss (Respondent 4)  Diets: There are quite a few gaps in [understanding] how climate change is affecting the accessibility (i.e. affordability) and distribution of healthy diets (i.e. HIC diets impact LMIC livelihoods). Furthermore, there is a gap in understanding how climate change impacts vary by population intersections (e.g. gender, age, rural-urban residence). There is well-established research on models and suggested dietary patterns (e.g. Planetary Health Diet) for climate change mitigation, but more research is needed with real-world experiments to examine the actual feasibility of adopting such diets across the global population, especially due to the uneven impacts of climate change on the food system. More work is underway in these areas but not yet published (Respondents 4, 5, 8). |
| --- |

### Supplemental Results 4: Methodological recommendations made by experts

| **NOTE: These consultation responses are presented as direct quotation or faithful summary of statements made by respondents (numbered to maintain anonymity).**  Research focus, methods and training: Overall, there is gap in integrating all dimensions of the FSNH nexus. There are gaps on both agriculture to nutrition interactions, and more importantly the added interactions of this dynamic from climate change. “Most of the literature focuses on fairly simple pathways (e.g. heat stress affecting food production) rather than more complex interactions such as climate change compounding other drivers which affect agriculture, nutrition and health, or having unanticipated outcomes” (Respondent 9).  Advancing work on any of the outlined evidence gaps requires funding opportunities for this specific area of research, interdisciplinary training for junior scholars, and support for research that integrates the perspectives of key stakeholders, from local communities to political actors (Respondents 3, 4, 6, 8). “Mechanisms of influence on ANH outcomes require a lot of specialist knowledge to develop, apply and validate” (Respondent 3). Progress “requires collaboration across lots of disciplines and scales” (Respondent 8). Even given proper data and perfect models, there will need to be “supporting capacities to use the tools, realize value around tools” and enable decision-making (Respondent 6).  Lastly, several respondents mentioned a lack of research focus on LMIC, for instance even in the development and application of data resources and approaches (Respondents 2, 4, 10 and 12). “LCA remains a strongly European and North American led approach that is really lacking application and validation in LMICs” (Respondent 12).  Data: “Currently we are not collecting the right kind of data, there are few open-access and independent data sets available [to answer the most pressing questions]. Lots of tools are developed but then are not publicly available, not institutionalised, or they are not collecting data as part of mandate. Data collected should be used for decision-making.” (Respondent 6). “Thus, there is a big push to collect metrics at various scales and of different dimensions of food systems, including nutrition and health (particularly driven by IFPRI, CGIAR and AgMIP). We need to continue this push and explore how some of those metrics could be projected into the future and related to climate change. There is a need for funders, policy makers and private sector incumbents to come together to work improve data collection and accessibility.” (Respondent 8).  Models: To reduce uncertainty in nutrition impact models in any significant way will require systematic and targeted model development, based on institutionalized funding support, similar to the climate modelling community. For instance, it is the industry standard for hundreds of full-time employees to maintain a climate model, but only a handful of PhD and post-doctorates develop crop models. There is a need for systematic long-term field experiments, harmonized model development and testing, and standardized model intercomparison projects across institutions and countries.  (Respondent 7, echoed by Respondents 5 and 6).  Tools: “More work is required to establish robust metrics and tools for integrating Indigenous and Local Knowledge (ILK) into FSNH research. Qualitative and quantitative evaluations of climate risk impacts on FSNH among LMICs should also be developed, particularly regarding agriculture adaption strategies.” (Respondent 4). “Models and tools that can compare impacts and trade-offs from different policies, programmes and actions” [would be transformative in the field] (Respondent 2). “As these tools are developed, at least some need to be open-source and user-friendly to maximise research uptake.” (Respondent 6). “There is a distinct lack of decision tools that are freely accessible, low-input and do not require extensive data knowledge available to policy-makers and government employees. [We need] ways to look at food systems in totality, and triggers for performance. We are not currently building tools that are relevant for governments” (Respondent 5). At present, there are “no correct surveillance and forecasting tools” available (Respondent 6).  There were also comments on the validity of common tools in this space: “True validation of individual and sets of indicators commonly used to proxy for the key dimensions of climate, planet, food system, health, economics, justice dimensions is still needed” (Respondent 10). |
| --- |

1. HLPE. 2020. Food security and nutrition: building a global narrative towards 2030. A report by the High Level Panel of Experts on Food Security and Nutrition of the Committee on World Food Security, Rome. [↑](#footnote-ref-1)
2. Pollock M, Fernandes RM, Becker LA, Pieper D, Hartling L. Chapter V: Overviews of Reviews. In: Higgins JPT, Thomas J, Chandler J, Cumpston M, Li T, Page MJ, Welch VA (editors). Cochrane Handbook for Systematic Reviews of Interventions version 6.4 (updated August 2023). Cochrane, 2023. Available from www.training.cochrane.org/handbook. [↑](#footnote-ref-2)
3. O’Neill J, Tabish H, Welch V, Petticrew M, Pottie K, Clarke M, et al. Applying an equity lens to interventions: using PROGRESS ensures consideration of socially stratifying factors to illuminate inequities in health. Journal of Clinical Epidemiology. 2014 Jan 1;67(1):56–64. [↑](#footnote-ref-3)
4. Please see the Cochrane Website [here](https://methods.cochrane.org/cmi/overviews-reviews#:~:text=Cochrane%20Overviews%20of%20Reviews%20(Overviews,their%20results%20across%20important%20outcomes.) [↑](#footnote-ref-4)
5. Please see the EU Commission website [here](https://knowledge4policy.ec.europa.eu/publication/food-systems-definition-concept-application-un-food-systems-summit-paper-scientific_en). [↑](#footnote-ref-5)
